# Quantitative bias analysis in practice: review of software for regression with unmeasured confounding

**DOI:** 10.1186/s12874-023-01906-8

**Published:** 2023-05-04

**Authors:** Emily Kawabata, Kate Tilling, Rolf H. H. Groenwold, Rachael A. Hughes

**Affiliations:** 1 MRC Integrative Epidemiology Unit, University of Bristol, Bristol, UK; 2 Population Health Sciences, Bristol Medical School, University of Bristol, Bristol, UK; 3 Department of Clinical Epidemiology, Leiden University Medical Center, Leiden, The Netherlands; 4 Department of Biomedical Data Sciences, Leiden University Medical Center, Leiden, The Netherlands

**Keywords:** Causal inference, Quantitative bias analysis, Sensitivity analysis, Software review, Unmeasured confounding

## Abstract

**Background:**

Failure to appropriately account for unmeasured confounding may lead to erroneous conclusions. Quantitative bias analysis (QBA) can be used to quantify the potential impact of unmeasured confounding or how much unmeasured confounding would be needed to change a study’s conclusions. Currently, QBA methods are not routinely implemented, partly due to a lack of knowledge about accessible software. Also, comparisons of QBA methods have focused on analyses with a binary outcome.

**Methods:**

We conducted a systematic review of the latest developments in QBA software published between 2011 and 2021. Our inclusion criteria were software that did not require adaption (i.e., code changes) before application, was still available in 2022, and accompanied by documentation. Key properties of each software tool were identified. We provide a detailed description of programs applicable for a linear regression analysis, illustrate their application using two data examples and provide code to assist researchers in future use of these programs.

**Results:**

Our review identified 21 programs with $62\%$ created post 2016. All are implementations of a deterministic QBA with $81\%$ available in the free software R. There are programs applicable when the analysis of interest is a regression of binary, continuous or survival outcomes, and for matched and mediation analyses. We identified five programs implementing differing QBAs for a continuous outcome: treatSens, causalsens, sensemakr, EValue, and konfound. When applied to one of our illustrative examples, causalsens incorrectly indicated sensitivity to unmeasured confounding whereas the other four programs indicated robustness. *sensemakr* performs the most detailed QBA and includes a benchmarking feature for multiple unmeasured confounders.

**Conclusions:**

Software is now available to implement a QBA for a range of different analyses. However, the diversity of methods, even for the same analysis of interest, presents challenges to their widespread uptake. Provision of detailed QBA guidelines would be highly beneficial.

**Supplementary Information:**

The online version contains supplementary material available at 10.1186/s12874-023-01906-8.

## Background

The main aim of many epidemiology studies is to estimate the causal effect of an exposure on an outcome (here onward, shortened to exposure effect). In observational studies participants are not randomised to exposure (or treatment) groups. Consequently, factors that affect the outcome are typically unevenly distributed among the exposure groups, and a direct comparison between the exposure groups will likely be biased due to confounding. Standard adjustment methods (such as standardization, inverse probability weighting, regression adjustment, g-estimation, stratification and matching) assume the adjustment model is correct and a sufficient set of confounders has been measured without error [1]. Failure to appropriately account for unmeasured or poorly measured confounders in analyses may lead to invalid inference [2–4].

There are several approaches to assess causality which depend on assumptions other than “no unmeasured confounding” (e.g., self-controlled study designs, prior event rate ratio, instrumental variable analysis, negative controls, perturbation variable analysis, and methods that use confounder data collected on a study sub-sample [5]). When none of these approaches are applicable (e.g., study lacks an appropriate instrument or sub-sample data on the unmeasured confounders) then the analyst must assess the sensitivity of the study’s conclusions to the assumption of no unmeasured confounding using a quantitative bias analysis (QBA; also known as a sensitivity analysis). A QBA can be used to quantify the potential impact of unmeasured confounding on an exposure effect estimate or to quantify how much unmeasured confounding would be needed to change a study’s conclusions.

Currently, QBA methods are not routinely implemented. A recent study published in 2016 found that the use of QBA for unmeasured confounding had not increased in the years $2010-2012$ compared to the $2004-2007$ period [6]. Lack of knowledge about QBA, and of analyst-friendly methods and software have been identified as barriers to the widespread implementation of a QBA [7–9]. In the past decade, there have been several reviews of QBA methods [2, 5, 9–18]. Of these, three papers reviewed software implementations of QBA methods: [18] gave an overview of QBA to unmeasured confounding and a tutorial on the newly released R package *tipr* [19], the supplementary of [13] provided a brief summary of software implementing Rosenbaum-style QBA methods [20], and [11] reviewed software implementations before its publication in July 2014. Also, comparisons of QBA methods have primarily been limited to analyses with a binary outcome [10, 21–28].

The aim of our software review was to identify the latest available software to conduct a QBA to unmeasured confounding and to describe the key properties of each software program. Note that, we focused on QBA to unmeasured confounding caused by a study not collecting data on these confounders as opposed to mismeasurement of measured confounders. We then describe, illustrate and compare QBA software applicable when the analysis of interest is a linear regression. We illustrate how to apply these methods using a real-data example from the Barry Caerphilly Growth (BCG) study [29, 30], and we also illustrate and provide R and Stata code implementing these methods when applied to publicly-accessible data from the $2015-2016$ National Health and Nutrition Examination Survey (NHANES) study [31] (see Additional files 1, 2 and 3).

### Quantitative bias analysis for unmeasured confounding

We want to estimate the effect of an exposure (or treatment) *X* on an outcome *Y*. The $Y-X$ association is confounded by measured covariates *C* and unmeasured confounders *U*. The naive estimate of the exposure effect, $\hat{\beta }_{X|C}$, assumes no unmeasured confounding and is estimated by controlling for *C* only.

We can use a QBA to quantify the likely magnitude and direction of the bias, due to unmeasured confounding, under different plausible assumptions about *U* (assuming no other sources of bias). Generally, a QBA requires a model (known as a bias model) for the observed data, *Y*, *X* and *C*, and unmeasured data, *U*. The bias model will include one or more parameters (known as bias or sensitivity parameters) which cannot be estimated from the observed data. Therefore, values for these bias parameters must be prespecified before conducting the QBA. Typically, the bias parameters specify the strength of the association between *U* and *X* given *C*, and between *U* and *Y* given *X* and *C* [23]. Information about the likely values of these bias parameters may be obtained from external sources (such as external validation studies, published literature, or expert opinion) [8], and from benchmarking (also known as calibration) where strengths of associations of measured covariates *C* with *X* and *Y* are used as benchmarks [32] for the bias parameters. We shall denote the bias parameters by $\phi$ and the bias-adjusted estimate of the exposure effect assuming $\phi$ by $\hat{\beta }_{X|C,U(\phi )}$.

There are two broad classes of QBA methods: deterministic and probabilistic [7]. A deterministic QBA specifies a range of values for each bias parameter of $\phi$ and then calculates $\hat{\beta }_{X|C,U(\phi )}$ for all combinations of the prespecified values of $\phi$. Typically, the results are displayed as a plot or table of $\hat{\beta }_{X|C,U(\phi )}$ against different values of $\phi$. In contrast, a probabilistic QBA specifies a prior probability distribution for $\phi$ to explicitly model the analyst’s assumptions about which combinations of $\phi$ are most likely to occur and to incorporate their uncertainty about $\phi$ [7, 24]. Averaging over this probability distribution generates a distribution of estimates of $\hat{\beta }_{X|C,U(\phi )}$ which is summarised to give a point estimate (i.e., the most likely $\hat{\beta }_{X|C,U(\phi )}$ under the QBA’s assumptions) and an interval estimate (i.e., defined to contain the true exposure effect with a prespecified probability) which accounts for uncertainty due to the unmeasured confounding and sampling variability [7].

A QBA is often conducted as a tipping point analysis, where the analyst identifies the values of $\phi$ that correspond to a change in the study conclusions (known as the “tipping point”). A tipping point analysis may be applied to the point estimate or confidence interval (CI) of the exposure effect; for example, to identify the values of $\phi$ corresponding to a null effect, or the values of $\phi$ corresponding to a statistically insignificant effect of a non-null point estimate. If the values of $\phi$ at the tipping point(s) are considered unlikely then the study conclusions are said to be robust to unmeasured confounding.

## Methods

### Software review

We conducted a systematic review of publicly available software implementations of QBA described in articles published between 1st January 2011 and 31st December 2021 (inclusive), and listed their key properties. We defined “software” to be either a web tool with a user-interface or software code that (i) was not specific to a particular data example (i.e., we excluded examples of code from empirical analyses that required code adaptation before application to another example), (ii) was freely available to download in January 2022, and (iii) was accompanied by documentation detailing the software’s features.

Our literature search was conducted in three stages. In stage 1, we used Web of Science to identify papers that mentioned “quantitative bias analysis” and “unmeasured confounding” (or their synonyms) in either the title, abstract or as keywords (see Supplementary Box 1 in Additional file 1). In stage 2, the abstracts were reviewed by two independent reviewers to determine if they were eligible for data extraction with any disagreements resolved by consensus. Eligible abstracts were published articles that either introduced a new QBA method or software implementation, compared or reviewed existing QBA methodology, or gave a tutorial on QBA. Examples of ineligible abstracts were meeting abstracts, commentaries, articles where a QBA was not conducted but mentioned as further work, and articles solely focused on answering applied questions (and so included limited information on the statistical methodology used). In stage 3, we extracted from the full text information about the analysis of interest, the QBA method, and the software used to implement the QBA.

### Illustration of QBA software applicable for a linear regression analysis

From our software review, we identified those programs applicable when the analysis of interest is a linear regression of an unmatched study. For each program, we provide descriptions of the software and implemented QBA method.

We applied these QBA programs to data from the BCG and NHANES studies. For both examples, the naive analysis was the linear regression *Y*|*X*, *C* with binary exposure *X*. We used measured variables to represent the unmeasured confounders *U*. So, in effect our analyses examined the effect of not including certain confounders and we assumed that after adjustment for *U* and *C* there was no unmeasured confounding. In the BCG example, *U* represents a single unmeasured confounder, statistical significance was defined at the $5\%$ level and adjustment for *U* did not change the study conclusions. See Additional file 1 for the NHANES example where *U* represents multiple unmeasured confounders, statistical significance was defined at the $1\%$ level and adjustment for *U* did change the study conclusions.

When supported by the program, we calculated benchmark values for $\phi$ based on *C* and the bias-adjusted results when $\phi$ was set to (multiples of) the benchmark values corresponding to the “strongest measured covariate” (i.e., the covariate that had the strongest associations with *X* and *Y*).

As this is an illustrative example of applying a QBA to unmeasured confounding, we have ignored other potential sources of bias (such as missing data) and only considered a small number of measured covariates. We restricted our analyses to participants with complete data on *Y*, *X*, *C* and *U*.

#### Description of the BCG study

The BCG study is a follow-up of a dietary intervention randomised controlled trial of pregnant women and their offspring [29, 30]. Data were collected on the offspring (gestational age, sex, and 14 weight and height measures at birth, 6 weeks, 3, 6, 9 and 12 months, and thereafter at 6-monthly intervals until aged 5 years) and their parents (anthropometric measures, health behaviours and socioeconomic characteristics). When aged 25, these offspring were invited to participate in a follow-up study in which standard anthropometric measures were recorded. We refer to the offspring, later young adults in the follow-up study, as the study participants.

Our analysis was a linear regression of adult body mass index (BMI) at age 25 on being overweight at age 5 years (BMI $\ge 17.44$ kg/m$^2$ [33]). Measured covariates *C* were participant’s gestational age, sex, birth weight, and parents’ height and weight measurements. The strongest measured covariate was maternal weight. The unmeasured confounder *U* was a measure of childhood socioeconomic position (SEP) (paternal occupational social class based on the UK registrar general classification [34]) with $U=1$ for professional or managerial occupations, and $U=0$ otherwise.

## Results

### Software review

After excluding duplicates, our Web of Science search identified 780 papers (flowchart of the review shown in Supplementary Fig. 1 in Additional file 1). We excluded 24 meeting abstracts and editorials, 379 articles that did not conduct a QBA to unmeasured confounding, and 239 articles on applied analyses. Of the remaining 138, 29 articles referred to 21 publicly available software implementations of a QBA.

Table 1 summarises the key features of the 21 software programs in ascending date-order of creation. All 21 programs implement a deterministic QBA, with only eight programs publicly available before 2017, and 17 implemented in the free software environment R [35]. Seven programs implement a QBA applicable for a matched observational study, five for a mediation analysis, and nine for a standard regression analysis. Five of the seven programs for a matched analysis (*sensitivityCaseControl, sensitivitymw, sensitivitymv, sensitivityfull* and *submax*) implement related QBA methods [20, 36] but for different types of matched observational studies. For example, *sensitivitymw* is applicable to matched sets with one exposed subject and a fixed number of unexposed subjects, and *sensitivitymv* to matched sets with one exposed subject and a variable number of unexposed subjects. Also, *submax* and *sensitivityCaseControl* exploit effect modification and different definitions of a case of disease, respectively, to further evaluate sensitivity to unmeasured confounding. Among the programs for mediation analysis, *MediationSensitivityAnalysis* evaluates sensitivity to unmeasured confounding of the mediator-outcome relationship only, while the remaining programs can also evaluate sensitivity to unmeasured confounding of the exposure-mediator and exposure-outcome relationships.

**Table 1:** Software programs implementing a quantitative bias analysis to unmeasured confounding published 2011 to 2021

		Analysis of interest				Bias analysis			
Name (Year created)	Environment	Type of analysis	Outcome	Exposure	Mediator	No. bias parameters	Benchmarking	Graphical plot	Tipping point
*isa*[37, 38] (2011)	Stata	simple^a^	con^b^	bin^c^	−	2	yes	line	null effect, stat. sig.^d^
*gsa*[11, 39] (2012)	Stata	simple	bin, con	bin, con, cat^e^	−	2	yes	scatter	null effect, stat. sig.
*SensitivityCase- Control*[40, 41] (2012)	R	matched	bin	bin	−	1	no	no	none
*causalsens*[42, 43] (2013)	R	simple	con	bin	−	1	yes	line with CI^f^	null effect, stat. sig.
*mbsens*[44] (2014)	Stata	matched	bin	bin	−	1	no	no	none
*sensitivitymw*[45–47] (2014)	R	matched	con, integer	bin	−	1	no	no	none
*treatSens*[48–50] (2014)	R	simple	con	bin, con	−	2 [$+1$ for *p*(*U*)]^g^	yes	contour	null effect, stat. sig.
*sensitivitymv*[45, 46, 51] (2015)	R	matched	con, integer	bin	−	1	no	no	none
*EValue*^h^ (2017) [52–56]	R, Stata, Web tool	simple, meta-analysis	bin, con, TTE^i^	bin, con	−	2	no	line	null effect, stat. sig.
*rmpw*[57, 58] (2017)	R	mediation	bin, con, cat	bin	bin, con, cat	2 or 4	yes	contour	stat. sig.
*sensitivityfull*[45, 59, 60] (2017)	R	matched	con, integer	bin	−	1	no	no	no
*submax*[61] (2017)	R	matched	con, integer	bin	−	1	no	no	stat. sig.
*Umediation*[62] (2017)	R	mediation	bin, con	bin, con	bin, con	3 [$+1$ or 2 for *p*(*U*)]	no	line	stat. sig.
*konfound*[63, 64] (2018)	R, Stata, Web tool	simple	bin, con	bin, con	−	2	yes	bar, causal diagram	stat. sig.
*sensmediation*[65, 66] (2018)	R	mediation	bin, con	bin, con	bin, con	1	no	line with CI	null effect, stat. sig.
*sensitivityCalibration*[32, 67] (2018)	R	matched	con	bin	−	3	yes	line	stat. sig.
*sensemakr*[68–70] (2019)	R, Stata, Web tool	simple	con	bin	−	2	yes	contour	null effect, stat. sig.
*ui*[71] (2019)	R	simple	bin	bin	−	2	no	line with CI and UI	null effect, stat. sig.
*mediationsens*[17, 72] (2020)	R	mediation	con, bin	bin	con, bin	2 or 3 [$+1$ for *p*(*U*)]	yes	contour	null effect, stat. sig.
*survsens*[73, 74] (2020)	R	simple	TTE	bin	−	2 [$+1$ for *p*(*U*)]	no	contour	null effect, stat. sig.
*MediationSensitivity- Analysis*[75] (2021)	Web tool	mediation	con	bin, con, cat	con	2	no	contour	null effect, stat. sig.

^a^ estimation of total effect of exposure in a sample of unmatched, independent observations from a single study; ^b^continuous variable; ^c^ binary variable; ^d^ statistical significance; ^e^ categorical variable; ^f^ CI: confidence interval; ^g^ option to set the parameter of the marginal distribution of *U*; ^h^ R package EValue, Stata command evalue and web tool E-value calculator; ^i^ time to event variable

Most programs require the outcome (of the analysis of interest) to be either binary or continuous. However, program *survsens* implements a QBA specifically for a Cox proportional hazards regression analysis and is applicable for survival outcomes with or without competing risks. All programs can be applied to a binary exposure and seven programs are also applicable to a continuous or categorical exposure. Also, all programs allow the analysis of interest to adjust for measured covariates *C* of any variable type and generally assume that *U* represents the part of the unmeasured confounder(s) that is independent of *C*. Nine programs use the measured covariates to calculate benchmark values for the bias parameters. The bias parameters represent the strength of the relationships between *U* and the exposure, outcome, or mediator. Programs *treatSens*, *Umediation*, *mediationsens*, and *survsens* also allow the analyst to vary the parameters of the marginal distribution of *U* (e.g., for binary *U* the probability $\Pr (U=1)$). Otherwise, these marginal parameters are set to a default value (e.g., $\Pr (U=1)=0.5$).

Almost all programs report the values of the bias parameters at prespecified tipping points. Also, most programs output the bias-adjusted results (e.g., point estimate, CI or P-value for the exposure effect) at prespecified values of the bias parameter(s) (exceptions include *isa*, *gsa*, *konfound*, and R and Stata implementations of *EValue*). Note that, programs *uMediation* and *ui* summarise the bias-adjusted results using uncertainty intervals, which incorporates uncertainty about the values of the bias parameters and sampling variability. Fifteen programs generate a graphical plot of their QBA results.

Two programs also implement a QBA to other sources of bias: *MediationSensitivtyAnalysis* can assess sensitivity to measurement error of the mediatior, outcome and measured covariates, and *EValue* can assess sensitivity to differential misclassification of an outcome or exposure and to sample selection bias. Furthermore, both programs can simultaneously assess sensitivity to multiple sources of bias.

### Illustration of QBA software applicable for a linear regression analysis

We describe and illustrate the following programs from Table 1 applicable for an unmatched analysis, where the exposure is binary and the exposure effect is estimated by a linear regression model: *treatSens* [48, 49], *causalsens* [42], *sensemakr* [76], *EValue* [52], and *konfound* [63]. For reasons of brevity, we excluded programs *isa* and *gsa* as they are similar to the more recently published *treatSens*.

Note that all five programs can be applied when $\hat{\beta }_{X|C}$ is not null, irrespective of whether $\hat{\beta }_{X|C}$ is statistically significant or not, and when $\hat{\beta }_{X|C}$ is null. However, for *treatSens* the tipping point for the point estimate is fixed at the null, and so this feature can only be used when $\hat{\beta }_{X|C}$ is not null.

Table 2 contains a summary of the five programs and immediately below we present the results from applying these programs to the BCG study. See Additional file 1 for detailed descriptions of the five programs, our application of the five programs to the NHANES example (accompanying R and Stata code in Additional files 2 and 3, respectively), and screenshots of the web tool implementations of *sensemakr*, *EValue* and *konfound*.

**Table 2:** Brief descriptions of the quantitative bias analysis software applicable for a linear regression analysis

***treatSens*** **Bias parameters: **$\phi =(\zeta ^Y,\zeta ^X)$ where $\zeta ^Y$ and $\zeta ^X$ represent the coefficients of *U* from regressions *Y*|*X*, *C*, *U* and *X*|*C*, *U*, respectively. **Benchmarks:** For each covariate $C_j$ of *C*, the benchmarks for $\zeta ^Y$ and $\zeta ^X$ are the coefficients of $C_j$ from regressions *Y*|*X*, *C* and *X*|*C*, respectively. **Method:** For prespecified values of $\phi =(\zeta ^Y,\zeta ^X)$, simulates *U* from model for joint distribution *X*, *Y*, *U*|*C* and then fits linear regression *Y*|*X*, *C*, *U* to the observed data and the simulated *U* to obtain $\hat{\beta }_{Y|X,C,U(\phi )}$ and its standard error. **Output:** Contour plot of $\hat{\beta }_{Y|X,C,U(\phi )}$ for different combinations of $\phi =(\zeta ^Y,\zeta ^X)$ with added benchmark values and indications of the values of $\phi =(\zeta ^Y,\zeta ^X)$ at the tipping points. Tabular outputs of the (1) values of $\phi =(\zeta ^Y,\zeta ^X)$ at the tipping points, (2) $\hat{\beta }_{Y|X,C,U(\phi )}$ and its standard error for prespecified values of $\phi =(\zeta ^Y,\zeta ^X)$, and (3) benchmark values for $\phi =(\zeta ^Y,\zeta ^X)$. **Other:** Standardises continuous variables to facilitate comparison between $\phi =(\zeta ^Y,\zeta ^X)$ and their benchmark values.
***causalsens*** **Bias parameters: **$\phi =(R_\alpha ^2)$ where the magnitude of $R_\alpha ^2$ represents the proportion of unexplained variance in the potential outcomes of *Y* (to non-exposure and exposure) that is explained by *U* and the sign of $R_\alpha ^2$ represents the direction of bias due to unmeasured confounding (e.g., if *U* explains $5\%$ of the unexplained variance, set $R_\alpha ^2=\pm 5\%$ to allow for bias towards and away from the null). **Benchmarks:** Based on partial $R^2$ of *Y* with each measured covariate. **Method:** Generates a modified outcome, $Y^{adj}_\phi$, adjusted for the bias due to unmeasured confounding using: (1) estimated probabilities $Pr(X=1|C)$ and a user-defined function (called the “confounding function”) for the average difference in the potential outcomes of *Y* between the exposure groups. Exposure effect results from regression $Y^{adj}_\phi |X,C$ are $\hat{\beta }_{Y|X,C,U(\phi )}$, and its confidence interval (CI). **Output:** Line plot of $\hat{\beta }_{Y|X,C,U(\phi )}$ and its CI for prespecified values of $R_\alpha ^2$ with each benchmark added as a positive and negative value (e.g., $\pm 5\%)$.
***sensemakr*** **Bias parameters: **$\phi =(R^2_{X \sim U|C},R^2_{Y \sim U|X,C})$ where $R^2_{X \sim U|C}$ is the proportion of the variance of *X*, not explained by *C*, that is explained by *U* and $R^2_{Y \sim U|X,C}$ is the proportion of the variance of *Y*, not explained by *X* and *C*, that is explained by *U*. **Benchmarks:** Calculates “benchmark bounds” for $R^2_{X \sim U|C}$ and $R^2_{Y \sim U|X,C}$ based on partial $R^2$ values of each measured covariate with *X* and *Y*, respectively. **Method:** (1) Formulae to calculate summary measures$^a$ for the point estimate and its t-value (called “robustness measures”). (2) Formulae to estimate $\hat{\beta }_{Y|X,C,U(\phi )}$ and its t-value for prespecified values of $\phi =(R^2_{X \sim U|C},R^2_{Y \sim U|X,C})$. **Output:** (1) Robustness values for the point estimate and its t-value at prespecified tipping points. (2) Contour plots of $\hat{\beta }_{Y|X,C,U(\phi )}$ and its t-value for different combinations of $\phi =(R^2_{X \sim U|C},R^2_{Y \sim U|X,C})$. Plots indicate values of $R^2_{X \sim U|C}$ and $R^2_{Y \sim U|X,C}$ that correspond to tipping points and (multiples of) the benchmark bounds. Also, outputs a table of bias-adjusted results, $\hat{\beta }_{Y|X,C,U(\phi )}$ and its CI, when $R^2_{X \sim U|C}$ and $R^2_{Y \sim U|X,C}$ equal (or equal multiples of) their benchmark bounds.
***EValue*** **Bias parameters: **$\phi =(RR_{XU},RR_{UY})$ where (for binary *X*, *Y* and single, binary *U*), $RR_{XU}$ and $RR_{UY}$ denote risk ratios for *X* on *U* and *U* on *Y* (conditional on *C*), respectively. **Benchmarks:** None provided. **Method:** Formulae to calculate summary measures$^a$ (called “E-values”) for the point estimate and CI limit. **Output:** Line plots indicating the combinations of $\phi =(RR_{XU},RR_{UY})$ at which $\hat{\beta }_{Y|X,C,U(\phi )}$ and its CI limit equate to their tipping point values with the E-values added to these plots. **Other:** Applicable for effect measures other than the risk ratio [52] and $\phi =(RR_{XU},RR_{UY})$ is defined for a single or multiple unmeasured confounders of type continuous, categorical or mixed [77].
***konfound*** **Bias parameters: **$\phi =(r_{X \sim U|C},r_{Y \sim U|C})$ where $r_{X \sim U|C}$ and $r_{Y \sim U|C}$ denote the partial correlation of *U* with *X* and *Y*, respectively, conditional on *C*. **Benchmarks:** Based on partial correlations of each covariate $C_j$ of *C* with *X* and *Y* given the remaining covariates. **Method:** Formulae to calculate: (1) “percent bias” the minimum percentage of $\hat{\beta }_{Y|X,C}$ explained by *U* at which the P-value for $\hat{\beta }_{Y|X,C,U(\phi )}$ equals statistical significance. (2) Summary measure$^a$ (called “impact threshold”). **Output:** Reports percent bias depicted as a bar graph and impact threshold depicted as a causal-type diagram. Stata command outputs a table of benchmark values for $\phi =(r_{X \sim U|C},r_{Y \sim U|C})$. **Other:** Percent bias and impact threshold evaluate how much unmeasured confounding would be needed to invalidate inference (i.e., change from statistically significant $\hat{\beta }_{Y|X,C}$ to statistically insignificant $\hat{\beta }_{Y|X,C,U(\phi )}$) or sustain inference (i.e., change from statistically insignificant $\hat{\beta }_{Y|X,C}$ to statistically significant $\hat{\beta }_{Y|X,C,U(\phi )}$).
a: For $\phi =(\phi _1,\phi _2)$, summary measure represents the minimum value of $\phi$ (when $\phi _1=\phi _2$) at the prespecified tipping point value.

#### Results from analysing the BCG study

Of the 951 individuals invited to the participate in the follow-up study, complete data for *X* (childhood overweight), *Y* (adult BMI), *C* (gestational age, sex, birth weight and parents’ height and weight measurements) and *U* (childhood SEP) were available for 542 participants. The naive estimate, $\hat{\beta }_{X|C}$, was 2.21 kg/m$^2$ ($95\%$ CI 1.30, 3.11 kg/m$^2$) and the fully adjusted estimate (i.e., adjusted for *C* and *U*) was 2.19 kg/m$^2$ ($95\%$ CI 1.29, 3.09 kg/m$^2$). Also, the coefficient of *U* from the linear regression *Y*|*X*, *C*, *U* was $-0.66$ kg/m$^2$ ($95\%$ CI $-1.57, 0.25$ kg/m$^2$) and the coefficient of *U* from the logistic regression *X*|*C*, *U* was $-0.23$ ($95\%$ CI $-0.85, 0.35$). Statistical significance was defined at the $5\%$ level.

We applied programs *treatSens*, *causalsens*, *sensemakr*, *EValue*, and *konfound* to data from the BCG study. For *treatSens* we used Probit regression for its treatment model because *X* was binary, for *causalsens* we used the one-sided confounding function because we assumed the exposure effect was the same in both exposure groups, and for *EValue* we calculated benchmark E-values by omitting one measured covariate at a time. We begin with a description of the outputted results from each program and then compare the results across the five programs.

##### *treatSens*

**Fig. 1:**
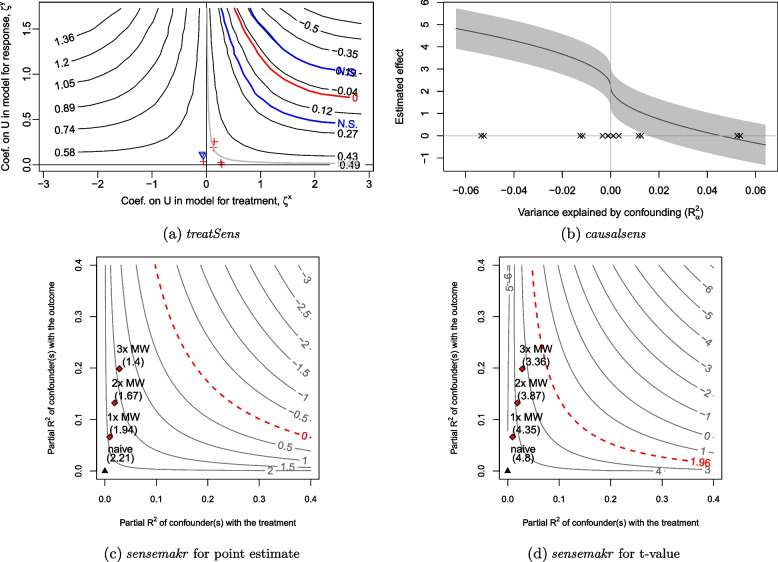
Quantitative bias analysis for child overweight on adult body mass index (Barry Caerphilly Growth study). Red contour (null effect in (**a**) and (**c**), t-value at $5\%$ significance in (**d**)), blue contours (bracket $5\%$ statistically insignificant estimates), black contour or line (bias-adjusted estimates), grey shaded area ($95\%$ confidence intervals for bias-adjusted estimates), pluses, inverted triangles, crosses, and diamonds (benchmarks using maternal weight (MW)), and black triangle (naive estimate)

Program *treatSens* outputs a contour plot (Fig. 1(a)) where each contour represents the different combinations of $\phi =(\zeta ^Y,\zeta ^X)$ that result in the same bias-adjusted estimate, $\hat{\beta }_{X|C,U(\phi )}$. For example, $\hat{\beta }_{X|C,U(\phi )}=0.43$ standard deviations of BMI (SD-BMI; or equivalently $\hat{\beta }_{X|C,U(\phi )}=1.93$ kg/m$^2$) when $\zeta ^Y=0.15$ and $\zeta ^X=1.00$, and when $\zeta ^Y=1.00$ and $\zeta ^X=0.15$. (Note that, *treatSens* standardises all continuous variables.) The black horizontal contour at $\zeta ^Y=0$ denotes the naive estimate of 0.49 SD-BMI (i.e., $\hat{\beta }_{X|C}=2.21$ kg/m$^2$), the red contour represents the combinations of $\phi$ that would result in a null exposure estimate, and the blue contours bracket statistically insignificant exposure estimates. The pluses and inverted triangles denote the benchmark values of $\phi$ based on measured covariates *C*: pluses represent covariates positively associated with adult BMI, and the inverted triangles represent covariates negatively associated with adult BMI with those negative associations rescaled by $-1$. The red cross furthest away from the origin denotes the strongest measured covariate (maternal weight).

##### *causalsens*

Program *causalsens* outputs a line plot (Fig. 1(b)) where the black line represents the bias-adjusted exposure estimates, the grey shaded area represents the corresponding $95\%$ CIs, and the crosses denote the benchmark values for $\phi =(R_{\alpha }^2)$ with each benchmark appearing twice to allow for both directions of effect. Values of $R_{\alpha }^2>0$ implies that individuals in the unexposed group tended to be healthier (i.e., lower adult BMI) than those in the exposed group even if everyone was of normal weight (or overweight) at age 5; and the converse for $R_{\alpha }^2<0$.

##### *sensemakr*

Program *sensemakr* outputs four contour plots: Fig. 1 (c) and (d) show the contour plots for the exposure effect estimate and its t-value, respectively, generated under the assumption that accounting for *U* moves the exposure effect estimate closer to the null, and Supplementary Fig. 2(a) and (b) (Additional file 1) show the same contour plots generated under the converse assumption. The contours have a similar interpretation as discussed for *treatSens*. For example, the red contour represents different combinations of $\phi =(R^2_{X \sim U|C}, R^2_{Y \sim U|X,C})$ that result in a null exposure effect (Fig. 1(c)) and the critical t-value corresponding to $5\%$ statistical significance (Fig. 1(d)). The black triangle denotes the naive estimate, $\hat{\beta }_{X|C}$, and the red diamonds denote once, twice and thrice the benchmark bounds based on the strongest measured covariate.

The robustness values for $\hat{\beta }_{X|C}$ and its t-value were $18.76\%$ and $11.56\%$, respectively. Thus, *U* would need to explain at least $18.76\%$ (or $11.56\%$) of the residual variance of both childhood overweight and adult BMI for the exposure effect adjusted for *C* and *U* to be null or in the reverse direction (or strictly positive but statistically insignificant).

##### *EValue*

The E-value for $\hat{\beta }_{X|C}$ and its lower CI limit were 2.50 and 1.93, respectively. Thus, if the associations between *U* and adult BMI and childhood overweight were at least 2.50 (or 1.93), on the risk ratio scale, then the exposure effect adjusted for *C* and *U* may be null or in the reverse direction (or strictly positive but statistically insignificant). Supplementary Fig. 3 (Additional file 1) shows the combinations of $\phi =(RR_{UY},RR_{XU})$ that correspond to a null bias-adjusted estimate (red contour) and a strictly positive but statistically insignificant bias-adjusted estimate (black contour).

##### *konfound*

The percent bias was $59.11\%$, depicted in the bar-graph shown in Supplementary Fig. 4 (Additional file 1), and the impact threshold was 0.13 with bias parameters $r_{X \sim U|C}=r_{Y \sim U|C}=\sqrt{0.13}$, depicted in the causal diagram shown in Supplementary Fig. 5 (Additional file 1). Therefore, in order for the exposure effect to be statistically insignificant after adjustment for *C* and *U* then (1) *U* would need to account for at least $59.11\%$ of $\hat{\beta }_{X|C}$ (i.e., $\hat{\beta }_{X|C,U(\phi )}\le 0.90$ kg/m$^2$), and (2) the partial correlations of *U* with adult BMI and child overweight must both exceed 0.36.

##### *Comparison of the results*

Table 3 summarises the bias-adjusted results of each program in scenarios where the associations between *U* and adult BMI and child overweight were half, once and twice as strong as the corresponding associations with the strongest measured covariate (i.e., $\phi$ set to 0.5, 1 and 2 $\times$ benchmark values for maternal weight).

**Table 3:** $\hat{\beta }_{X|C,U(\phi )}$ [$95\%$ confidence interval] when $\phi$ equals multiples of benchmark values for maternal weight (MW)

	$\hat{\beta }_{X|C,U(\phi )}\ [95\%$ confidence interval] in $kg/m^2$			
If $\phi$ set to	*treatSens*	*causalsens*	*sensemakr*	*konfound*
Bias towards the null				
$0.5 \times$ benchmark values of MW	2.19 [1.29, 3.09]	0.52 $[-0.39, 1.42]$	2.08 [1.18, 2.97]	[excludes 0]
$1 \times$ benchmark values of MW	2.14 [1.24, 3.04]	$-0.19$ $[-1.09, 0.72]$	1.94 [1.06, 2.82]	[excludes 0]
$2 \times$ benchmark values of MW	1.91 [1.04, 2.78]	$-1.18$ $[-2.08, -0.28]$	1.67 [0.82, 252]	[excludes 0]
Bias away from the null				
$0.5 \times$ benchmark values of MW	2.24 [1.34, 3.14]	3.90 [3.00, 4.80]	2.34 [1.45, 3.23]	[excludes 0]
$1 \times$ benchmark values of MW	2.31 [1.42, 3.21]	4.60 [3.70, 5.51]	2.47 [1.60, 3.35]	[excludes 0]
$2 \times$ benchmark values of MW	2.54 [1.67, 3.41]	5.59 [4.69, 6.50]	2.74 [1.89, 3.59]	[excludes 0]

Considering unmeasured confounding towards or away from the null, if *U* was comparable to the strongest measured covariate (with respect to its associations with adult BMI and child overweight) then *treatSens* and *sensemakr* report that adjusting for *C* and *U* would give similar results to those of the naive analysis and *konfound* indicates the exposure effect would remain statistically significant. Also, *sensemakr*’s robustness values were substantially higher than the benchmark bounds for $R^2_{X \sim U|C}$ and $R^2_{Y \sim U|X,C}$ even when these benchmarks were based on all of *C* (Supplementary Table 1 in Additional file 1). Similarly, the benchmark E-values when omitting the strongest measured covariate and *U* were comparable to the E-values when omitting *U* only (Supplementary Table 2 in Additional file 1), indicating that the exposure effect adjusted for *C* and *U* would remain above the null and statistically significant. Furthermore, *treatSens*, *sensemakr*, and *konfound* indicate that *U* would need to be more than double the strength of the strongest measured covariate in order to change the study conclusions (i.e., a null or doubling of the exposure effect, or a statistically insignificant effect). Conversely, *causalsens* suggests adjusting for *U* comparable to the strongest measured covariate could result in an exposure effect close to the null or more than double the naive estimate.

Provided the naive analysis included all of the important confounders then it seems unlikely that the confounding effect of *U*, childhood SEP, could be more than twice as strong as the strongest measured covariate, especially given that childhood SEP would likely be correlated with at least some of the measured covariates. Therefore, under these assumptions, *treatSens*, *sensemakr*, *konfound*, and *EValue* indicates robustness of the BCG study conclusions to unmeasured confounding by childhood SEP which was inline with the fully adjusted results. In contrast, *causalsens* suggested study conclusions could differ if we were able to adjust for childhood SEP.

## Discussion

We have conducted an up-to-date review of software implementations of QBA to unmeasured confounding, and a detailed illustration of the latest software applicable for a linear regression analysis of an unmatched study.

### Remarks on the software review

All programs implement a deterministic QBA, and most are available in the free software environment R. The majority were developed in the latter half of the past decade and include programs available when the naive analysis is a mediation analysis, meta-analysis and a survival analysis. Many programs include features such as benchmarking and graphical displays of the QBA results to aid interpretation.

A limitation of our review was that we focused on software described in the published literature, in particular between 2011 and 2021 (inclusive). Consequently, our review excluded unpublished software implementations and example-specific software code of QBAs (which may explain the absence of probabilistic QBAs in our review). Our reasoning for focusing on published literature was to provide the reader with a certain amount of confidence regarding the quality of the software (i.e., due to the peer-review process). Additionally, we focused on software implementations that do not require any programming adaptions to encourage the uptake of QBAs among all users irrespective of their programming skills. We recognise that additional software programs are available such as other implementations of QBA methods discussed in this review (e.g., another implementation of the E-value [78]), published software before 2011 (e.g., Stata command *episens* [79]), QBA methods published before 2011 (e.g., Axelson et al [80], R package *episensr* [81] implementing a QBA method published in 2009 [7]), and programs of other QBA methods (e.g., *TippingSens* [82]).

### Remarks on the comparison of software applicable for a linear regression analysis

Our illustrative example showed that even QBA software applicable to the same naive analysis can implement distinct QBA methods. All programs were straightforward to implement and instantly generated the results except for *treatSens* which took about 10 minutes to run when applied to a moderately-sized dataset (see the NHANES example in Additional file 1). All programs provided information about the amount of unmeasured confounding at the tipping points; however, *treatSens*, *sensemakr* and *causalsens* also provided information on the bias-adjusted results for any specified level of unmeasured confounding with minimal extra burden to the analyst.

Out of the five programs we compared *sensemakr* performs the most detailed QBA. It generates bias-adjusted results for prespecified levels of unmeasured confounding (similarly to *treatSens* and *causalsens*), reports a summary measure at prespecified tipping points (similarly to *EValue* and *konfound*) and conducts a QBA in a worse-case scenario of unmeasured confounding (similarly to *EValue*). Program *EValue* implements a flexible QBA which can be applied to a wide range of effect measures and makes minimal assumptions about the unmeasured confounding (e.g., allows *U* to be a modifier of the $X-Y$ relationship). However, the downside of this flexibility is that the analyst may be unaware of the additional assumptions required when converting their effect measure to the risk ratio scale and it can be challenging to establish plausible values for its bias parameters (either from external data or from benchmarking). Also, a notable limitation of programs *EValue* and *konfound* is that they are restricted to establishing robustness to unmeasured confounding (i.e., cannot provide results adjusted for likely levels of unmeasured confounding) and *konfound* only considers sensitivity to changes in statistical significance. The upside of the programs’ simplicity is that they require only summary data and so can be easily applied to multiple published studies, with the *EValue* extended to random-effects meta analyses [55]. Three strengths of *treatSens* over the other programs are: (1) its imputation-style QBA method will be familiar to many analysts, (2) its bias parameters (i.e., regression coefficients) are more likely to be reported by published studies than the bias parameters of the other programs (e.g., partial $R^2$ values), and (3) *treatSens* can also be applied when the analysis of interest is a non-parametric model (Bayesian additive regression tree). A potential weakness of *treatSens* is that it simulates *U* from a limited choice of joint distributions.

We compared software programs applicable when the analysis of interest is a linear regression since previous comparisons of QBA methods have primarily focused on analyses of binary outcomes [10, 21–28]. Of the software we compared, programs *konfound* and *EValue* can be applied to a binary outcome, with *EValue* also applicable when the exposure effect is a hazard ratio. Future work could compare QBA methodology for analyses of other types of outcomes such as survival and categorical outcomes.

### QBA with benchmarking

Several programs in our review provided benchmark values to aid interpretation of the QBA results. Note that, *sensemakr* can provide benchmark bounds for its bias parameters based on a group of measured covariates which provides a useful aid when considering multiple unmeasured confounders. One noted issue with benchmarking is that the benchmarks tend to be based on the naive models, *Y*|*X*, *C* and *X*|*C*, and do not adjust for the omission of *U* [32, 68]. See Cinelli and Hazelett for a discussion on why ignoring *U* can affect the benchmark values even when *U* is assumed to be independent of *C* [68]. Examples of QBAs using benchmarking that accounts for the omission of *U* include *sensemakr*, [32], and [83].

### Multiple unmeasured confounders

Examples of QBAs tend to focus on a single unmeasured confounder when in fact many weaker unmeasured confounders can jointly change a study’s conclusions [4]. However, several QBA methods are generalisable to multiple unmeasured confounders without burdening the analyst with additional bias parameters. For example, a common assumption is that *U* represents a linear combination of multiple unmeasured confounders, with the elementary scenario that *U* is a single unmeasured confounder. A drawback of this appealing assumption is that the QBA tends to be conservative for multiple unmeasured confounders [68]. Alternatively, a QBA method may leave the functional form of *U* unspecified and instead define its bias parameters as upper bounds (such as the *EValue* where *U* is a categorical variable with categories representing all possible combinations of the multiple unmeasured confounders and its bias parameters $RR_{XU}$ and $RR_{UY}$ are the maximum risk ratios comparing any two categories of *U* [77]). A drawback of these upper bounds is that they correspond to extreme situations, making it hard to locate appropriate benchmark values or external information. To address both drawbacks, a QBA could explicitly model each unmeasured confounder separately whilst allowing for correlations between the confounders, although this would then increase the number of bias parameters. If many unmeasured confounders are suspected, then the analyst should question if a QBA is suitable since the accuracy of a QBA generally relies on a study having measured key confounders. Importantly, a QBA is not a replacement for a correctly designed and conducted study.

### Deterministic and probabilistic QBAs

Our review did not identify any publicly available software implementations of probabilistic QBAs published between 2011 and 2021 (inclusive). In part this may be due to the perception that probabilistic QBAs are more difficult to apply than deterministic QBAs (e.g., needing to choose probability distributions for the bias parameters) and the misconception that probabilistic QBAs require specialist software for Bayesian inference [7]. Note that, a probabilistic QBA with a uniform distribution on the bias parameters is equivalent to a deterministic QBA [7]. Although a deterministic QBA can suffice to demonstrate robustness or sensitivity of inferences (e.g., when a study has measured all known confounders) [8], a probabilistic approach has several key advantages: (1) allows the user to specify that some values of the bias parameter(s) are more likely than others, (2) the results can be summarised in a format familiar to epidemiologists (i.e., a point estimate and corresponding interval estimate), (3) the interval estimate gives a more accurate representation of the total uncertainty in a QBA (i.e., uncertainty about the bias parameters and uncertainty due to random sampling), and (4) for a QBA with more than two bias parameters a probabilistic approach can be more practicable than a deterministic approach due to the challenges of presenting and interpreting the results when there are a large number of possible value combinations of the bias parameters [7]. Further work is needed to provide published software implementations of probabilistic QBAs.

### Concluding remarks

In summary, there have been several new software implementations of deterministic QBAs, most of which are available in R. Deterministic QBAs are often interpreted as tipping point analyses with statistical significance as one of the tipping points. Given the call to move away from reliance on statistical significance [84], we recommend QBA software that provide bias-adjusted results for all specified values of the bias parameters to give a complete picture of the effect of unmeasured confounding (such as *treatSens*, *sensemakr* and *causalsens*). Our comparative evaluation has illustrated the wide diversity in the types of QBA method that can be applied to the same substantive analysis of interest. Such diversity of QBA methods presents challenges in the widespread uptake of QBA methods. Guidelines are needed on the appropriate choice of QBA method, along with greater availability of software implementations of probabilistic QBAs and in platforms other than R.

## Supplementary Information

**Additional file 1.**

**Additional file 2.**

**Additional file 3.**

## Data Availability

The Barry Caerphilly Growth study dataset analysed during the current study is available from Prof. Y. Ben-Shlomo (University of Bristol) but restrictions apply to the availability of these data, which were used under license for the current study, and so are not publicly available. Data are however available from the authors upon reasonable request and with permission of Prof. Y. Ben-Shlomo. The National Health and Nutrition Examination Survey dataset analysed during the current study is available in the NHANES Questionnaires, Datasets, and Related Documentation repository, https://wwwn.cdc.gov/nchs/nhanes/Default.aspx. All software programs are freely available as detailed in their documentation (see references).

## Abbreviations

BCG: Barry Caerphilly Growth
BMI: Body Mass Index
CI: Confidence Interval
MW: Maternal Weight
NHANES: National Health and Nutrition Examination Survey
QBA: Quantitative Bias Analysis
SEP: Socio-Economic Position
SD: Standard Deviation
