# Supplementary material for: Quantitative bias analysis in practice: review of software for regression with unmeasured confounding
Source: BMC Med Res Methodol. 2023 May 4;23:111. doi: 10.1186/s12874-023-01906-8 (PMC10158211; doi:10.1186/s12874-023-01906-8)
Supplement: Supplementary file 1 — Additional file 1. [file 12874_2023_1906_MOESM1_ESM.pdf]

Supplementary Materials

Quantitative bias analysis in practice: Review of software for regression with unmeasured confounding

E Kawabata[1,2], K Tilling[1,2], RHH Groenwold[3,4], and RA Hughes[1,2]

[1]MRC Integrative Epidemiology Unit, University of Bristol, Bristol, United Kingdom
[2]Population Health Sciences, Bristol Medical School, University of Bristol, Bristol, United Kingdom
[3]Department of Clinical Epidemiology, Leiden University Medical Center, Leiden, The Netherlands
[4]Department of Biomedical Data Sciences, Leiden University Medical Center, Leiden, The Netherlands

Contents

1 Software review **3**

2 Descriptions of the software implementing a quantitative bias analysis for linear regression **5**
2.1 *treatSens* 5
2.2 *causalsens* 7
2.3 *sensemakr* 8
2.4 *EValue* 10
2.5 *konfound* 12

3 The Barry Caerphilly Growth study **14**
3.1 Screenshots of the web tools 19

4 The National Health and Nutrition Examination Survey **23**
4.1 Description of the study 23
4.2 Results 24
4.3 Screenshots of the web tools 32

1 Software review

Supplementary Box 1: Web of Science search terms for software review. See `https://webofscience.help.clarivate.com/en-us/Content/search-operators.html` and `https://webofscience.help.clarivate.com/en-us/Content/search-rules.htm` on how to interpret these terms which are specific to the Web of Science Core Collection.
(TS=(unmeasured NEAR/3 confound*) OR TS=(unmeasured NEAR/3 variable*) OR TS=(unmeasured NEAR/3 covariate*) OR TS=(unmeasured NEAR/3 factor*) OR TS=(unmeasured NEAR/3 predictor*) OR TS=(uncontrolled NEAR/3 confound*) OR TS=(uncontrolled NEAR/3 variable*) OR TS=(uncontrolled NEAR/3 covariate*) OR TS=(uncontrolled NEAR/3 factor*) OR TS=(uncontrolled NEAR/3 predictor*) OR TS=(omitted NEAR/3 confound*) OR TS=(omitted NEAR/3 variable*) OR TS=(omitted NEAR/3 covariate*) OR TS=(omitted NEAR/3 factor*) OR TS=(omitted NEAR/3 predictor*) OR TS=(omission NEAR/3 confound*) OR TS=(unobserved NEAR/3 confound*) OR TS=(unobserved NEAR/3 variable*) OR TS=(unobserved NEAR/3 covariate*) OR TS=(unobserved NEAR/3 factor*) OR TS=(unobserved NEAR/3 predictor*) OR TS=(hidden NEAR/3 confound*) OR TS=(hidden NEAR/3 variable*) OR TS=(hidden NEAR/3 covariate*) OR TS=(hidden NEAR/3 factor*) OR TS=(hidden NEAR/3 predictor*) OR TS=(selection NEAR/3 bias*) OR TS=(residual NEAR/3 confound*) OR TS=(hidden NEAR/3 bias*))<br>AND<br>(TS=(sensitivity NEAR/3 analy*) OR TS=(bias NEAR/3 analys*) OR TS=(bias NEAR/3 model*))<br>AND<br>(TS=(confound*))

Supplementary Figure 1: Flowchart of the software review

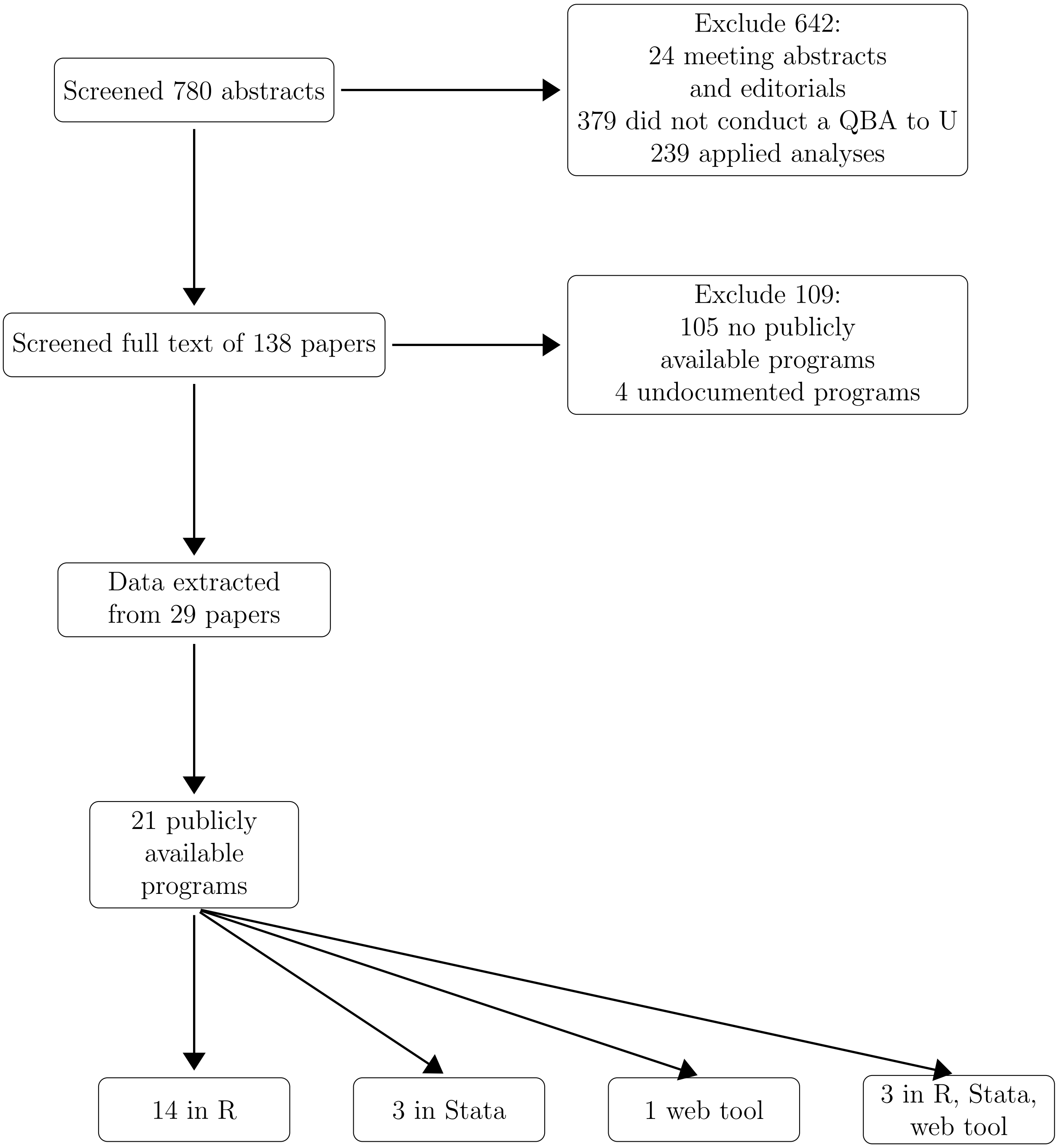

2 Descriptions of the software implementing a quantitative bias analysis for linear regression

In this section we give further details of the five quantitative bias analysis (QBA) programs summarised in Table 2 of the main manuscript. These programs implement a QBA to unmeasured confounding when the naive analysis is a linear regression model for an unmatched study.

2.1 *treatSens*

The program implements a simulated-based approach comparable to multiple imputation for missing data [1]. For prespecified values of bias parameters, ϕ, *treatSens* simulates U multiple times from the conditional distribution of $U|Y, X, C$ implied by the bias model. For each set of simulated values of U, the exposure effect is estimated from a linear regression of Y given X, C and the simulated U, and then Rubin's rules [1] are used to combine the multiple sets of results into a single estimate for $\hat{\beta}_{X|C,U(\phi)}$ and its standard error. Note that, U denotes the part of the unmeasured confounding that is independent of measured covariates C (i.e., U is independent of C). Also, U can represent a single unmeasured confounder or a linear combination of multiple unmeasured confounders.

The bias model is a joint model for $Y, X, U|C$ which consists of three sub-models: the analysis model (regression of Y given X, C and U), the treatment model (regression of X on C and U), and the marginal model for U (marginal distribution of U). When the naive analysis is a linear regression, *treatSens* offers two types of bias models depending on whether X is continuous or binary: (1) for continuous X, the bias model consists of linear regression $Y|X, C, U$ (analysis model), linear regression $X|C, U$ (treatment model) and standard normal distribution (marginal model for U), and (2) for binary X, the bias model consists of linear regression $Y|X, C, U$ (analysis model), probit regression $X|C, U$ (treatment model) and Bernoulli distribution (marginal model for U, default is $\Pr(U = 1) = 0.5$). Each bias model has two bias parameters $\phi = (\zeta^Y, \zeta^X)$: ζ^Y is the coefficient for U from the analysis model, $Y|X, C, U$, and ζ^X is the coefficient for U from the treatment model, $X|C, U$. To allow for bias in both directions (i.e., increased exposure effect, and reduced or reversed exposure effect), positive and negative values are specified for ζ^X. For fixed values of $\phi = (\zeta^Y, \zeta^X)$, the remaining coefficients of the treatment and analysis models are estimated from the observed data which are then used to derive the parameters of the model for $U|Y, X, C$

(implied by the bias model). See Carnegie et al for further details including the general algorithm used to derive these parameter estimates [2]. The coefficients of measured covariates C from regressions $Y|X, C$ and $X|C$ are used as benchmark values for ζ^Y and ζ^X, respectively [2]. All continuous variables are standardised (to have mean 0 and standard deviation of 1) to facilitate comparison between these benchmark values and the bias parameters.

For a fixed value of ϕ, the process of simulating U and then analysing the observed and simulated data is repeated K (≥ 2) times and the K sets of exposure estimates and corresponding standard errors $[\hat{\beta}^k_{X|C,U(\phi)}$, $se(\hat{\beta}^k_{X|C,U(\phi)})$ for $k = 1, 2, \ldots, K]$ are combined to generate the bias-adjusted estimate $\hat{\beta}_{X|C,U(\phi)} = \dfrac{1}{K}\sum_{k=1}^{K}\hat{\beta}^k_{X|C,U(\phi)}$ and its standard error

$se(\hat{\beta}_{X|C,U(\phi)}) = \sqrt{W + (1 + K^{-1})B}$ where $W = \dfrac{1}{K}\sum_{k=1}^{K}(se(\hat{\beta}^k_{X|C,U(\phi)}))^2$ reflects conventional sampling variance and the extra variance due to simulating U is given by $B = \dfrac{1}{K-1}\sum_{k=1}^{K}(\hat{\beta}^k_{X|C,U(\phi)} - (\hat{\beta}_{X|C,U(\phi)})^2)$. This whole process is repeated for different values of ϕ.

Program *treatSens* outputs a contour plot of the bias-adjusted estimates, $\hat{\beta}_{X|C,U(\phi)}$, for different combinations of ζ^Y and ζ^X, indicating the combinations of ζ^Y and ζ^X that correspond to tipping points for the point estimate (fixed at the null) and statistical significance (analyst can set the significance level; default is 5%). Additional outputs include tables of: (1) combinations of ζ^Y and ζ^X at the tipping points, (2) the K sets of exposure estimates and corresponding standard errors $[\hat{\beta}^k_{X|C,U(\phi)}$, $se(\hat{\beta}^k_{X|C,U(\phi)})$ for $k = 1, 2, \ldots, K]$ for all prespecified combinations of ζ^Y and ζ^X, and (3) benchmark values for ζ^Y and ζ^X.

Program *treatSens* is available as an R package from GitHub page `https://github.com/vdorie/treatSens` and it requires individual participant data. The analyst can use *treatSens* when the estimand of interest is the average treatment effect (ATE), the average treatment effect among the treated (ATT), or the average treatment effect among the controls (ATC). Other options include: (1) reparameterising the bias parameters as partial correlations instead of model coefficients (only for continuous X), (2) specifying multiple central processing unit cores for parallel processing to help speed-up the run-time of *treatSens*, (3) specifying the number of times U is simulated for each combination of ζ^Y and ζ^X (default is 20), and (4) specifying the marginal probability $\Pr(U = 1)$ (default is 0.5). Program *treatSens* has been extended to implement a semi-parametric QBA applicable when the

analysis model is a non-parametric Bayesian additive regression tree model [3, 4].

2.2 *causalsens*

The program generates a modified outcome, Y_{ϕ}^{adj}, which is adjusted for the bias due to unmeasured confounding for a prespecified value of ϕ [5]. The naive analysis is then refitted using Y_{ϕ}^{adj} instead of Y and the resulting exposure effect estimate and confidence interval (CI) are the bias-adjusted results.

The implemented QBA method is based on the potential outcomes framework [6]. Program *causalsens* requires a binary X, and so there are two potential outcomes per subject: $Y(0)$ when not exposed and $Y(1)$ when exposed. The bias model consists of a treatment model and a "confounding function" [7, 8]. The treatment model is a logistic regression used to estimate the probability of being in the exposed group given C. The confounding function, $f(x, C) = E[Y(x)|X = x, C] - E[Y(x)|X = 1-x, C]$, quantifies the average difference in potential outcomes $Y(x)$ (for $x = 0$ or 1) between those in the exposed and unexposed groups (conditional on C), with any nonzero difference attributed to unmeasured confounding [9]. Setting $f(x, C) = 0$ corresponds to the assumption of no unmeasured confounding. The confounding function is parameterised by a single bias parameter, α, which implies a combination of the strengths of the $X - U$ and $Y - U$ relationships, conditional on C. To allow for bias in both directions (i.e., increased exposure effect, and reduced or reversed exposure effect), positive and negative values are specified for α.

Program *causalsens* supplies two choices for the confounding function, named the "one-sided function" and the "alignment function", and also allows the analyst to specify their own function. The one-sided function, $f(X = x, C) = \alpha(2x - 1)$, assumes the true exposure effect is identical in the exposed and unexposed groups. When $\alpha > 0$ the mean of the potential outcomes to exposure (and non-exposure) is higher for the exposed group than the unexposed group, leading $\hat{\beta}_{X|C}$ to be positively biased; and vice versa for $\alpha < 0$ [9]. Under the alignment function, $f(X = x, C) = \alpha$, the true exposure effect differs between the exposure groups. Naive estimate $\hat{\beta}_{X|C}$ is biased by $\alpha(\Pr[X = 0|C] - Pr[X = 1|C])$, where $\Pr[X = 0|C]$ and $\Pr[X = 1|C]$ denote the probability of being unexposed and exposed (conditional on C), respectively [9]. When the same proportion of people are exposed and unexposed ($\Pr[X = 1|C] = \Pr[X = 0|C] = 0.5$) then $\hat{\beta}_{X|C}$ is unbiased by U. However, when proportionally more individuals are unexposed than exposed, conditional on C, (i.e., $\Pr[X = 0|C] > \Pr[X = 1|C]$) then $\hat{\beta}_{X|C}$ is positively biased for $\alpha > 0$ and negatively biased for $\alpha < 0$; and vice versa when pro-

portionally more individuals are exposed than unexposed, conditional on C, (i.e., $\Pr[X = 0|C] < \Pr[X = 1|C]$) [9].

Since bias parameter, α, is difficult to interpret, *causalsens* offers the alternative parameterisation, R^2_α, where the magnitude of R^2_α represents the proportion of unexplained variance in the potential outcomes of Y (to non-exposure and exposure) that is explained by U and the sign of R^2_α represents the direction of bias due to unmeasured confounding (e.g., $R^2_\alpha = \pm 5\%$ specifies that U explains 5% of the unexplained variance of the potential outcomes of Y and allows adjusting for U to move the point estimate towards and away from the null). See [5] for an explanation on how to derive R^2_α from α.

Program *causalsens* outputs a line plot and a table of $\hat{\beta}_{X|C,U(\phi)}$ and corresponding 95% CI for different values of ϕ. Additionally, *causalsens* reports benchmarks for R^2_α based on the partial R^2 of Y with each covariate in C. Note that, the line plot does not explicitly indicate the tipping points of the point estimate or CI. However, the analyst can easily determine from the plot the values of ϕ at which the bias-adjusted estimate, $\hat{\beta}_{X|C,U(\phi)}$, equals a specific value (e.g., the null), or the values of ϕ at which the CI includes or excludes the null.

Program *causalsens* is an R package available from GitHub page `https://github.com/mattblackwell/causalsens` or the Comprehensive R Archive Network (CRAN). It requires individual participant data and exposure X must be binary but outcome Y can be continuous or binary, although the naive analysis is restricted to a linear regression. Program *causalsens* can be applied when the estimand of interest is the ATE or ATT. The program outputs results with respect to the original bias parameter, α, and the alternative parameterisation, R^2_α. By default, *causalsens* chooses values for ϕ based on features of Y. The analyst can override this default setting to specify their own values and can customise their own confounding function. Note that, the R package downloaded from CRAN fixes the statistical significance to be at the 5% level whereas the R package downloaded from GitHub allows the analyst to change the statistical significance level.

2.3 *sensemakr*

The program uses formulae to estimate $\hat{\beta}_{X|C,U(\phi)}$ and its t-value for prespecified values of ϕ. Additionally, *sensemakr* reports summary measures, called "robustness values", which quantify the minimum amount of unmeasured confounding needed to change a study's conclusions, conditional on C [10].

The bias model has two bias parameters $R^2_{X\sim U|C}$ and $R^2_{Y\sim U|X,C}$: $R^2_{X\sim U|C}$ is the proportion of the variance of X, not explained by C, that is explained by U, and $R^2_{Y\sim U|X,C}$ is the proportion of the variance of Y, not explained by

X and C, that is explained by U. Considering both directions of effect of U, the bias-adjusted estimate is $\hat{\beta}_{X|C,U(\phi)} = \hat{\beta}_{X|C} \pm \widehat{\Delta}_\phi$, where $\widehat{\Delta}_\phi$ denotes the magnitude of the bias due to levels of unmeasured confounding specified by $\phi = (R^2_{X\sim U|C}, R^2_{Y\sim U|X,C})$ and is calculated as

$$|\widehat{\Delta}_\phi| = se(\hat{\beta}_{X|C})\sqrt{\frac{R^2_{Y\sim U|X,C}R^2_{X\sim U|C}}{1 - R^2_{X\sim U|C}} \times df}, \tag{1}$$

where $se(\hat{\beta}_{X|C})$ and df are the standard error of the exposure effect and degrees of freedom from the naive analysis, respectively. The standard error of the bias-adjusted estimate is calculated as

$$se(\hat{\beta}_{X|C,U(\phi)}) = se(\hat{\beta}_{X|C})\sqrt{\frac{1 - R^2_{Y\sim U|X,C}}{1 - R^2_{X\sim U|C}} \times \frac{df}{df - 1}}. \tag{2}$$

The robustness value for the point estimate (or t-value) represents the minimum value of $R^2_{X\sim U|C}$ and $R^2_{Y\sim U|X,C}$, when $R^2_{X\sim U|C} = R^2_{Y\sim U|X,C}$, such that $\hat{\beta}_{X|C,U(\phi)}$ (or its t-value) equals its prespecified tipping point value; for example, the null (or the 5% critical t-value). A robustness value close to 1 indicates that strong unmeasured confounding would be needed to change the study conclusions, whilst a value close to 0 indicates that very weak unmeasured confounding could change the conclusions. The robustness values are derived from the above equations by setting $R^2_{X\sim U|C} = R^2_{Y\sim U|X,C}$ and $\widehat{\Delta}_\phi$ to the relevant tipping point value.

Program *sensemakr* provides benchmark values (called "benchmark bounds") for $R^2_{X\sim U|C}$ and $R^2_{Y\sim U|X,C}$ based on the partial R^2 values of X and Y, respectively, with each measured covariate of C. Note that, these benchmark bounds are calculated adjusting for the omission of the unmeasured confounder(s) (i.e., these benchmark bounds are not simply partial R-squared values from regressions $X|C$ and $Y|X,C$) [10]. See [10] and [11] for a discussion on the disadvantages of ignoring U when deriving benchmark values, even when U is assumed to be independent of C. Additionally, *sensemakr* can calculate benchmark bounds based on a group of measured covariates.

Program *sensemakr* makes no distributional assumptions about U but does assume that U is either a single unmeasured confounder or a linear combination of two or more unmeasured confounders. Cinelli and Hazlett state that their assumption about multiple unmeasured confounders is conservative [10].

Program *sensemakr* outputs robustness values for the point estimate and t-value, and contour plots of $\hat{\beta}_{X|C,U(\phi)}$ and corresponding t-value for prespecified values of ϕ. These contour plots include markers representing multiples of the benchmark bounds (e.g., once, twice, or three times the magnitude of a benchmark bound) and indications of the combinations of $R^2_{X\sim U|C}$ and $R^2_{Y\sim U|X,C}$ that correspond to a tipping point for the point estimate or t-value. Also, *sensemakr* outputs a table of benchmark bounds and values of $\hat{\beta}_{X|C,U(\phi)}$ and corresponding CI when $R^2_{X\sim U|C}$ and $R^2_{Y\sim U|X,C}$ equal multiples of the benchmark bounds.

Program *sensemakr* is available as an R package (install from CRAN or GitHub page `https://github.com/carloscinelli/sensemakr`), Stata command (install from the Statistical Software Components (SSC) archive or GitHub page `https://github.com/resonance1/sensemakr-stata`), and as a web tool `https://carloscinelli.shinyapps.io/robustness_value/`. The analyst can either apply *sensemakr* to their individual participant data using R or Stata, or input summary data from the naive analysis using R or the web tool. By default, the direction of the effect of U is towards the null and the tipping points for the point estimate and t-value are the null and t-critical value at 5% statistical significance, respectively. Available options allow the analyst to set the direction of effect to be away from the null, a different statistical significance level, and a non-null value for the tipping point of the point estimate. Program *sensemakr* also implements a QBA for an extreme scenario in which all or most of the unexplained variation in Y is explained by U (e.g., $R^2_{Y\sim U|X,C} = 1$).

2.4 *EValue*

The program reports a summary measure, called an E-value, which quantifies the minimum amount of unmeasured confounding needed to change a study's conclusions, conditional on the measured covariates [12]. Here we describe the E-value when the tipping point of the point estimate is the null, although it can also be set to a non-null value (see supplementary materials of [12]).

The E-value is defined on the risk ratio scale and is derived from two bias parameters $\phi = (RR_{XU}, RR_{UY})$. For binary X and a single, binary U, RR_{XU} represents the risk ratio for the effect of X on U conditional on C and RR_{UY} represents the maximum risk ratio for the effect of U on Y conditional on C among the exposed and unexposed [13]. (Note that, U can represent a single or multiple unmeasured confounders of type continuous, categorical or mixed. See Ding and VanderWeele [13] for a general definition of RR_{XU} and RR_{UY}). A separate E-value is calculated for the point estimate and CI limit closest to the null (shortened to "CI limit" from here onward). The E-value

for the point estimate represents the minimum value of RR_{XU} and RR_{UY}, when $RR_{XU} = RR_{UY}$, such that the bias-adjusted point estimate is null or in the reverse direction to that of the naive point estimate. Similarly, the E-value for the CI limit represents the minimum value of RR_{XU} and RR_{UY}, when $RR_{XU} = RR_{UY}$, such that the exposure effect is no longer statistically significant after adjustment for C and U.

The E-value is a positive number ≥ 1 with higher values indicating that greater levels of unmeasured confounding (i.e., stronger $X - U$ and $Y - U$ associations) are required to change the study conclusions. When the naive point estimate is null (or its CI includes the null) the E-value for the point estimate (or CI limit) is 1, indicating that no unmeasured confounding is required to change the study conclusions. Importantly, the E-value is a measure of sensitivity to unmeasured confounding for a worst-case scenario (i.e., bias parameters RR_{XU} and RR_{UY} are set to values which maximise the bias due to unmeasured confounding) [14].

The E-value for the point estimate (CI limit) is calculated using the naive point estimate (CI limit from the naive analysis). When the exposure effect of interest is not a risk ratio, then the naive point estimate or CI limit must be converted to an approximate risk ratio before calculating the E-value [12]. For example, for standardised mean difference, $\hat{\beta}^{std}_{X|C}$, and corresponding standard error, $se(\hat{\beta}^{std}_{X|C})$, the approximate risk ratio for the point estimate is $\exp\{0.91 \times \hat{\beta}^{std}_{X|C}\}$ and the approximate risk ratio for a limit of the 95% CI is $\exp\{0.91 \times \hat{\beta}^{std}_{X|C} \pm 1.78 \times se(\hat{\beta}^{std}_{X|C}\})$ [12]. (See [12] for more details, including calculating approximate risk ratios for other types of effect measures.) For the remainder of this program description, we shall assume that the naive point estimate and naive CI limit are risk ratios (or approximate risk ratios).

The general formula for the E-value is

$$\text{E-value } = \widehat{RR} + \sqrt{\widehat{RR} \times (\widehat{RR} - 1)}, \tag{3}$$

where $\widehat{RR}$ denotes a risk ratio from the naive analysis and $\widehat{RR} > 1$. When the naive point estimate is greater than 1 (less than 1), the E-value for the point estimate is calculated by replacing $\widehat{RR}$ in equation (3) with the naive point estimate (inverse of the naive point estimate). Similarly, for a naive CI that excludes the null, when the point estimate is greater than 1 (less than 1), the E-value for the CI limit is calculated by replacing $\widehat{RR}$ with the lower limit of the naive CI (inverse of the upper limit of the naive CI).

The rationale of the E-value is based on bias factor BF_{ϕ} which represents the maximum relative amount the naive risk ratio can be altered by unmea-

sured confounding of a given strength specified by $\phi = (RR_{UY}, RR_{XU})$ [12]. This bias factor is defined as

$$BF_{\phi} = \frac{RR_{UY} RR_{XU}}{RR_{UY} + RR_{XU} - 1}. \tag{4}$$

According to VanderWeele and Ding [12], when the naive point estimate is greater than 1, dividing this naive point estimate by BF_{ϕ} gives a bias-adjusted point estimate for the maximum effect of unmeasured confounding of given strength $\phi = (RR_{UY}, RR_{XU})$ [12] (i.e., most attenuated risk ratio adjusted for C and U given ϕ). For a naive point estimate less than 1, multiply the naive point estimate by BF_{ϕ} to obtain the bias-adjusted point estimate. Similarly, for the CI limit.

Software to calculate an E-value is available as R package *EValue* [15, 16] (available from CRAN and from GitHub page `https://github.com/mayamathur/evalue_package`), Stata command *evalue* [17] (available from the SSC archive), and web tool the *e-value calculator* [15] (available at `https://www.evalue-calculator.com/evalue`). All implementations can be applied when the effect measure of interest is a risk ratio, risk difference, standardised mean difference, odds ratio or hazard ratio for a rare outcome (i.e., prevalence $< 15\%$), and odds ratio or hazard ratio for a common outcome (i.e., prevalence $\geq 15\%$). Also, the software only requires summary-level data (e.g., point estimate and CI limit from the naive analysis). The graphical output is a line plot of the values of RR_{UY} and RR_{XU} that correspond to a prespecified tipping point of the point estimate with the corresponding E-value indicated on the plot. Also, the Stata command, *evalue*, can add to the graphical output the equivalent line plot for the CI limit. Additionally, the *e-value calculator* includes a feature that outputs the bias factor, BF_{ϕ}, and corresponding bias-adjusted risk ratio for prespecified values of $\phi = (RR_{UY}, RR_{XU})$. Note that, program *EValue* does not supply benchmark values for RR_{UY} and RR_{XU}. For comparison purposes, VanderWeele and Ding [12] suggest omitting each measured covariate in turn and recalculating the E-value.

2.5 *konfound*

The program assesses sensitivity to a change in the statistical (in)significance status of $\hat{\beta}_{X|C}$ [18]. This includes the scenario where U explains away all of the statistical significance of $\hat{\beta}_{X|C}$ (i.e., $\hat{\beta}_{X|C}$ is statistically significant but $\hat{\beta}_{X|C,U(\phi)}$ is statistically insignificant) and the converse scenario where U restores the statistical significance of $\hat{\beta}_{X|C}$ (i.e., $\hat{\beta}_{X|C}$ is

statistically insignificant but $\hat{\beta}_{X|C,U(\phi)}$ is statistically significant). Program *konfound* refers to the first scenario as U "invalidating inference" and the second as U "sustaining inference". By default, the significance level is 5% and the null hypothesis is "no exposure effect", both of which can be changed by the analyst.

Program *konfound* reports two summary measures that quantify the minimum level of unmeasured confounding necessary to change conclusions on statistical significance: "percent bias" and "impact threshold". Percent bias is a measure of the minimum percentage of $\hat{\beta}_{X|C}$ that would need to be explained away by U in order for unmeasured confounding to invalidate or sustain inference [19, 20]. The formula for the percent bias is a function of estimated quantities from the naive analysis and the value of $\hat{\beta}_{X|C,U(\phi)}$ when its P-value is exactly κ% (for statistical significance defined at the κ% level). The impact threshold is also derived from estimated quantities of the naive analysis plus two bias parameters $\phi = (r_{X\sim U|C}, r_{Y\sim U|C})$: $r_{X\sim U|C}$ and $r_{Y\sim U|C}$ represent the partial correlation between U and X and between U and Y (conditional on C), respectively [21]. The impact threshold is the product $r_{X\sim U|C} \times r_{Y\sim U|C}$ when $r_{X\sim U|C}$ and $r_{Y\sim U|C}$ are equal and set to their minimum value such that statistical inference is invalidated or sustained. Note that, the percent bias measure is always positive but the impact threshold measure can be positive or negative depending on the direction of the correlation between U and X and between U and Y. For both measures, larger absolute values indicate greater robustness to unmeasured confounding.

Software *konfound* is available as an R package (from CRAN or GitHub page `https://github.com/jrosen48/konfound`), a Stata command (see [18] for download instructions), and a web tool `https://jmichaelrosenberg.shinyapps.io/konfound-it/`. The R and Stata implementations can be applied to individual participant data and to summary-level data using R function or Stata command *pkonfound*. The web tool and R package can be used with two types of summary-level data: (1) point estimate, standard error, sample size and number of measured covariates from the naive analysis, and (2) a 2×2 cross tabulation of exposure and outcome data.

The software outputs the percent bias (depicted by a bar graph, called a "threshold plot", when $\hat{\beta}_{X|C}$ is statistically significant) and the impact threshold (depicted by a causal-type diagram called a "correlation plot"; only generated by the R package and web tool). Only the Stata command provides benchmark values for $r_{X\sim U|C}$ and $r_{Y\sim U|C}$, which are the partial correlation of each measured covariate C_j with X and with Y, respectively, given the remaining measured covariates.

Extra features include a nonlinear option to conduct a QBA when the analysis model is a logistic or probit regression (although the R package and

Stata command report a warning to ignore the impact threshold for a binary outcome) and an R function and Stata command, called *mkonfound*, to apply the QBA to multiple studies.

3 The Barry Caerphilly Growth study

In this section, additional results of the QBA analysis of the Barry Caerphilly Growth study are presented.

Supplementary Figure 2: *sensemakr* contour plots when unmeasured confounding increases the exposure effect of child overweight on adult body mass index: black contours (bias-adjusted estimates for the point estimate in (a) and t-value at 5% significance in (b)), diamonds (benchmarks using maternal weight (MW)), and black triangle (naive estimate). Data from the Barry Caerphilly Growth study.

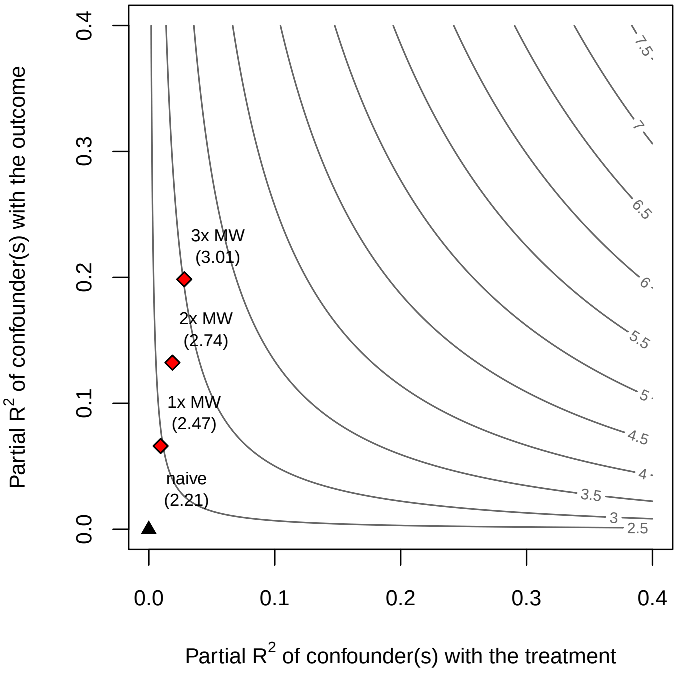

(a) *sensemakr* for point estimate

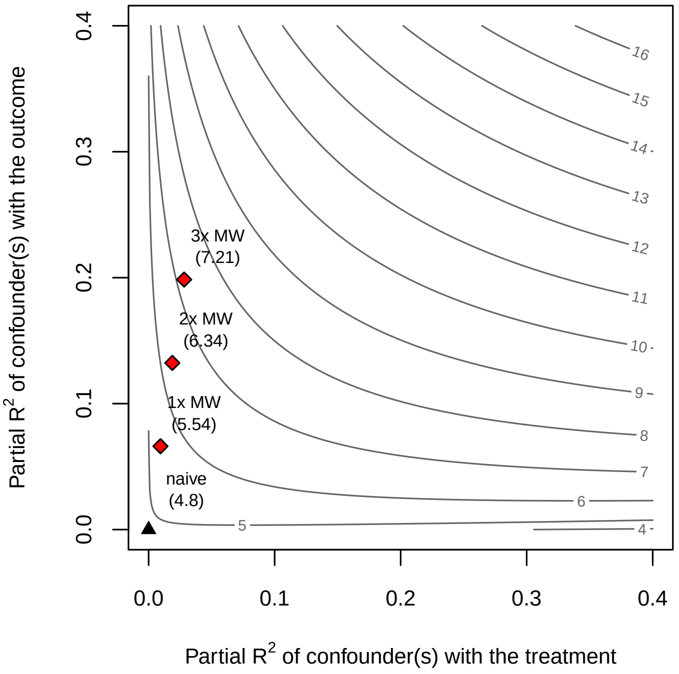

(b) *sensemakr* for t-value

Supplementary Table 1: Benchmark bounds for *sensemakr*'s bias parameters, $R^2_{X\sim U|C}$ and $R^2_{Y\sim U|X,C}$, based on partial R^2 of a measured covariate (or group of covariates) with exposure X (child overweight) and outcome Y (adult body mass index). Data from the Barry Caerphilly Growth study.

Measured covariate	$R^2_{X\sim U|C}(\%)$	$R^2_{Y\sim U|X,C}(\%)$
Gestational age	0.10	0.14
Sex	0.89	0.00
Birth weight	2.52	0.07
Paternal height	0.14	1.03
Paternal weight	0.73	3.77
Maternal height	0.27	1.43
Maternal weight	0.94	6.61
All covariates	5.47	13.52

Supplementary Figure 3: E-values for the effect of childhood overweight on adult body mass index from the Barry Caerphilly Growth study. Curves denote value combinations of the bias parameters that explain away 100% of the naive point estimate (red) and statistical significance at the 5% level (black); dots denote corresponding E-values.

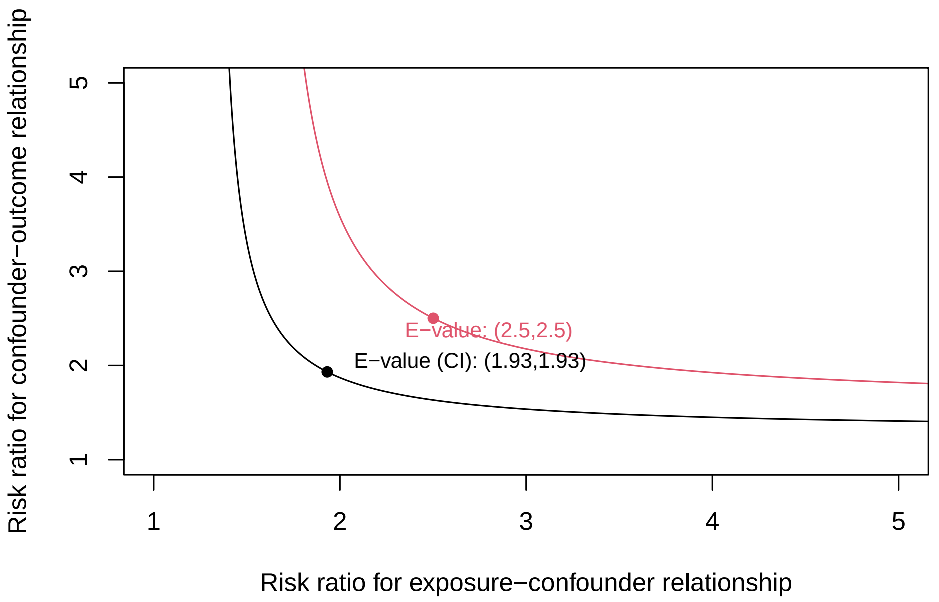

Supplementary Table 2: Benchmark E-values, for the point estimate and lower confidence interval (CI) limit for the effect of child overweight on adult body mass index from the Barry Caerphilly Growth study.

	E-value	
Measured covariate	Point estimate	Lower CI limit
Gestational age	2.49	1.92
Sex	2.50	1.94
Birth weight	2.53	1.96
Paternal height	2.53	1.95
Paternal weight	2.62	2.03
Maternal height	2.55	1.97
Maternal weight	2.68	2.08

Supplementary Figure 4: *konfound*: Threshold plot representing the percent bias necessary to invalidate inference (at 5% statistical significance) for the effect of childhood overweight on adult body mass index from the Barry Caerphilly Growth study.

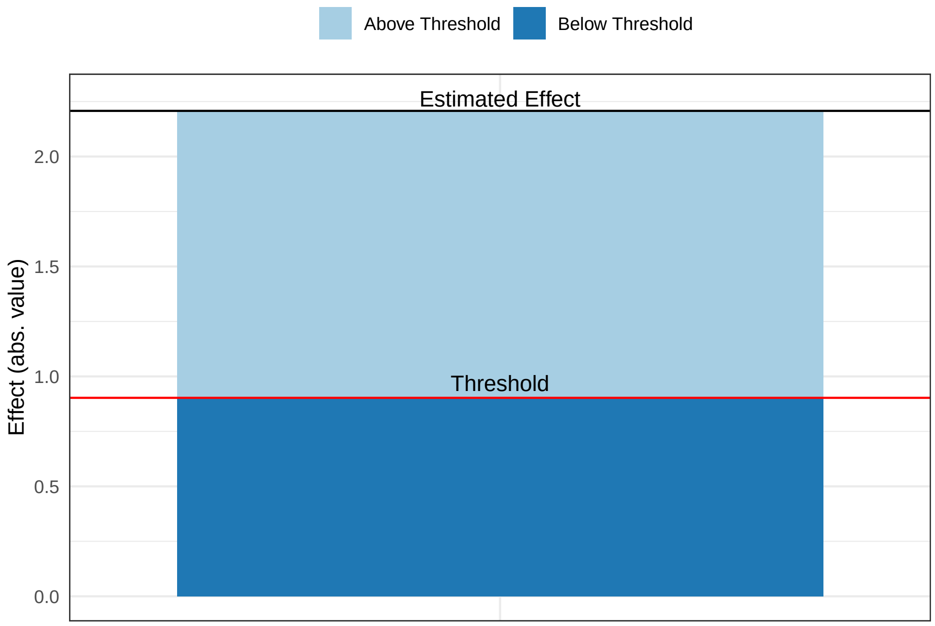

Supplementary Figure 5: *konfound*: Correlation plot of the impact of unmeasured confounding necessary to invalidate inference (at 5% statistical significance) for the effect of childhood overweight on adult body mass index from the Barry Caerphilly Growth study. $r_{X\sim U|C}$ and $r_{Y\sim U|C}$ denote partial correlations between unmeasured confounder U and exposure X, and between U and outcome Y, respectively, conditional on measured covariates C.

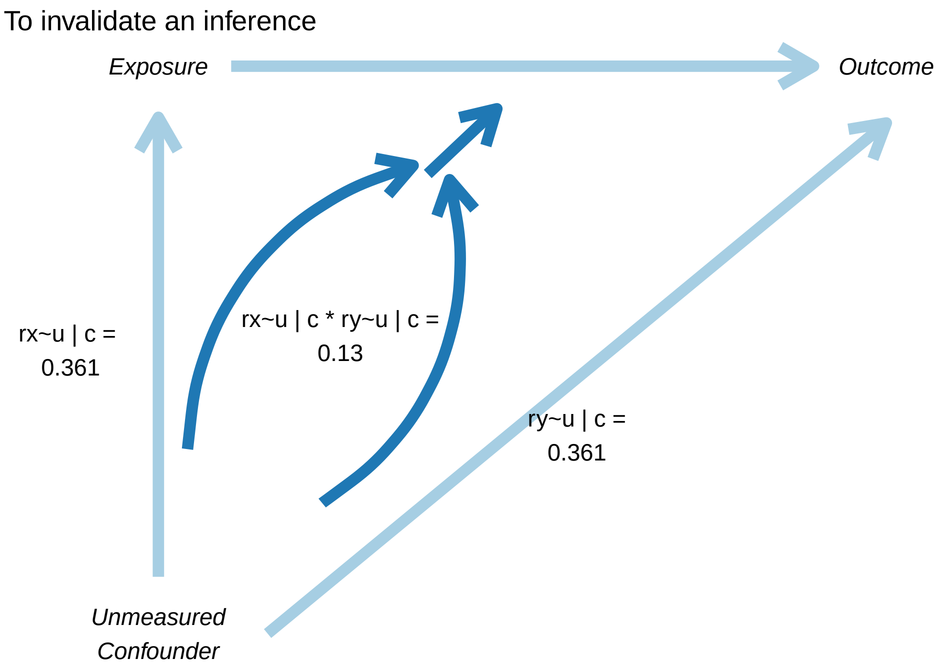

Supplementary Table 3: *konfound*: Benchmark values for $r_{X\sim U|C}$ and $r_{Y\sim U|C}$, and impact threshold $r_{X\sim U|C} \times r_{Y\sim U|C}$, based on partial correlations of each measured covariate with childhood overweight and adult body mass index. Data from the Barry Caerphilly Growth study.

Measured covariate	$r_{X\sim U\|C}$	$r_{Y\sim U\|C}$	Impact threshold
Gestational age	−0.0312	0.0298	−0.0009
Sex	0.0938	0.0216	0.0020
Birth weight	0.1568	0.0576	0.0090
Paternal height	−0.0368	−0.1062	0.0039
Paternal weight	0.0854	0.2030	0.0173
Maternal height	−0.0518	−0.1267	0.0066
Maternal weight	0.0965	0.2614	0.0252

3.1 Screenshots of the web tools

This section presents screenshots of the application of the web tools of *sensemakr*, *EValue*, and *konfound* to the Barry Caerphilly Growth study.

Supplementary Figure 6: Screenshot of the *sensemakr* web tool applied to the Barry Caerphilly Growth study.

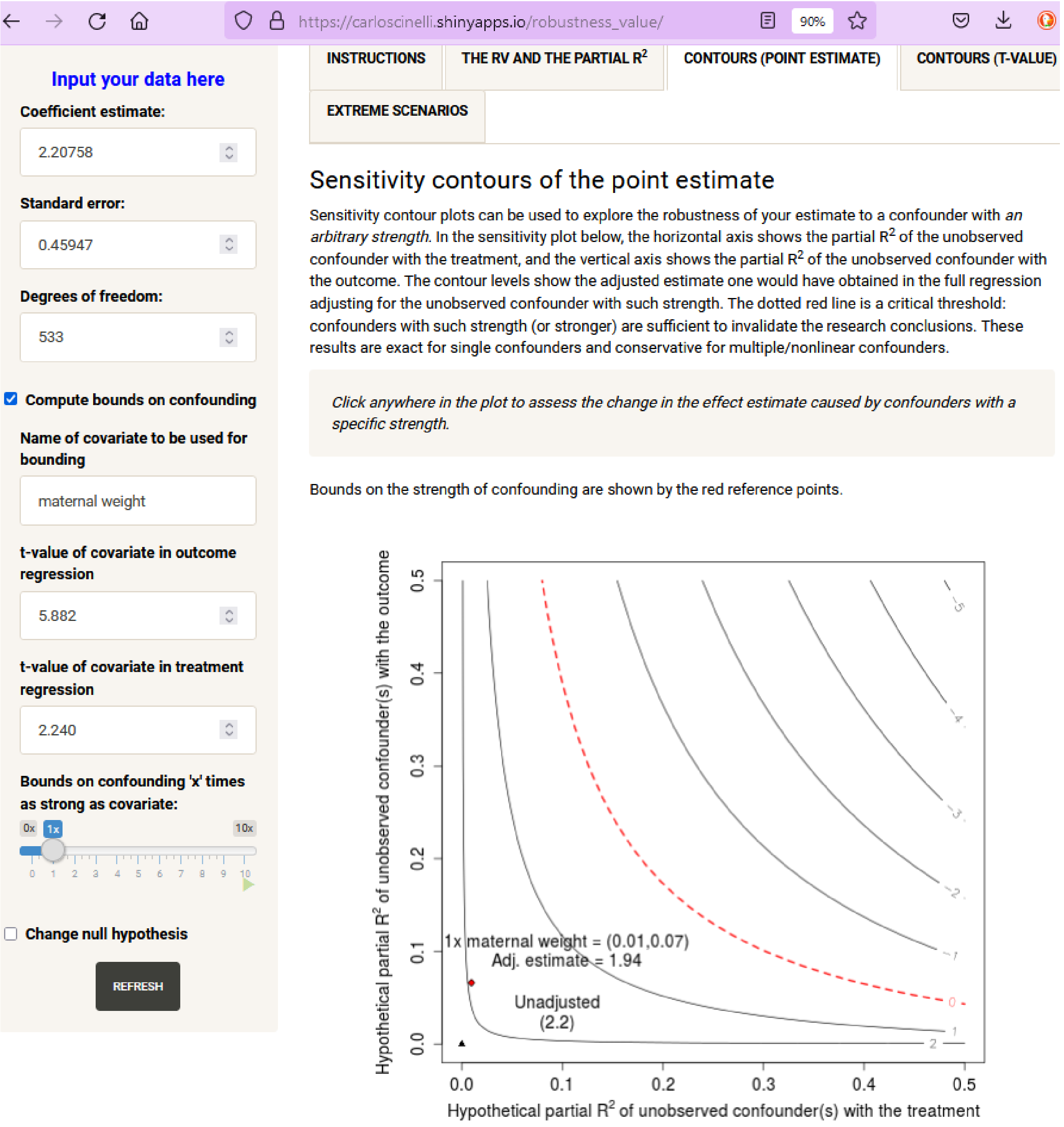

Supplementary Figure 7: Screenshot of the *e-value calculator* web tool applied to the Barry Caerphilly Growth study.

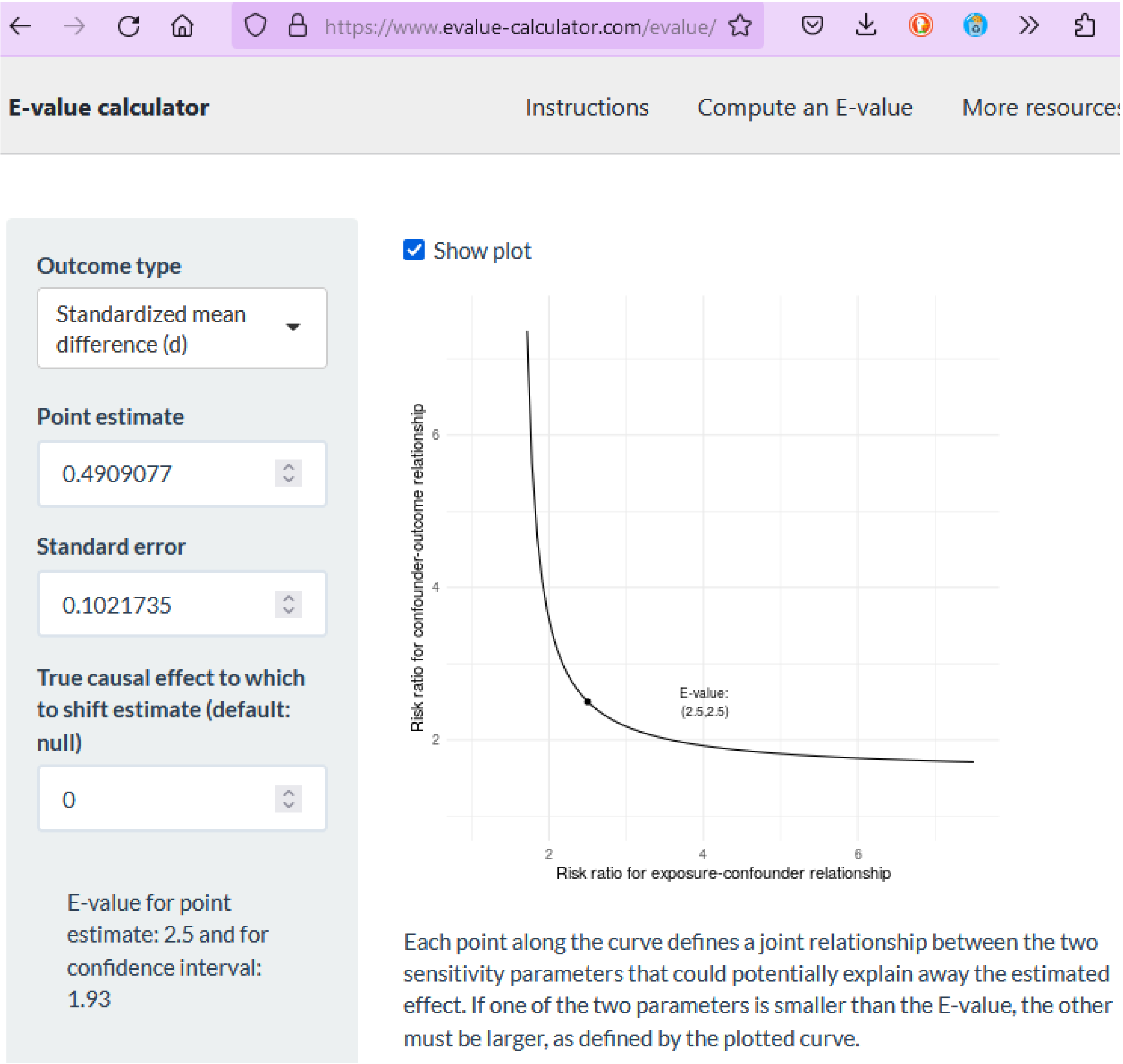

Supplementary Figure 8: Screenshot of the *konfound* web tool applied to the Barry Caerphilly Growth study.

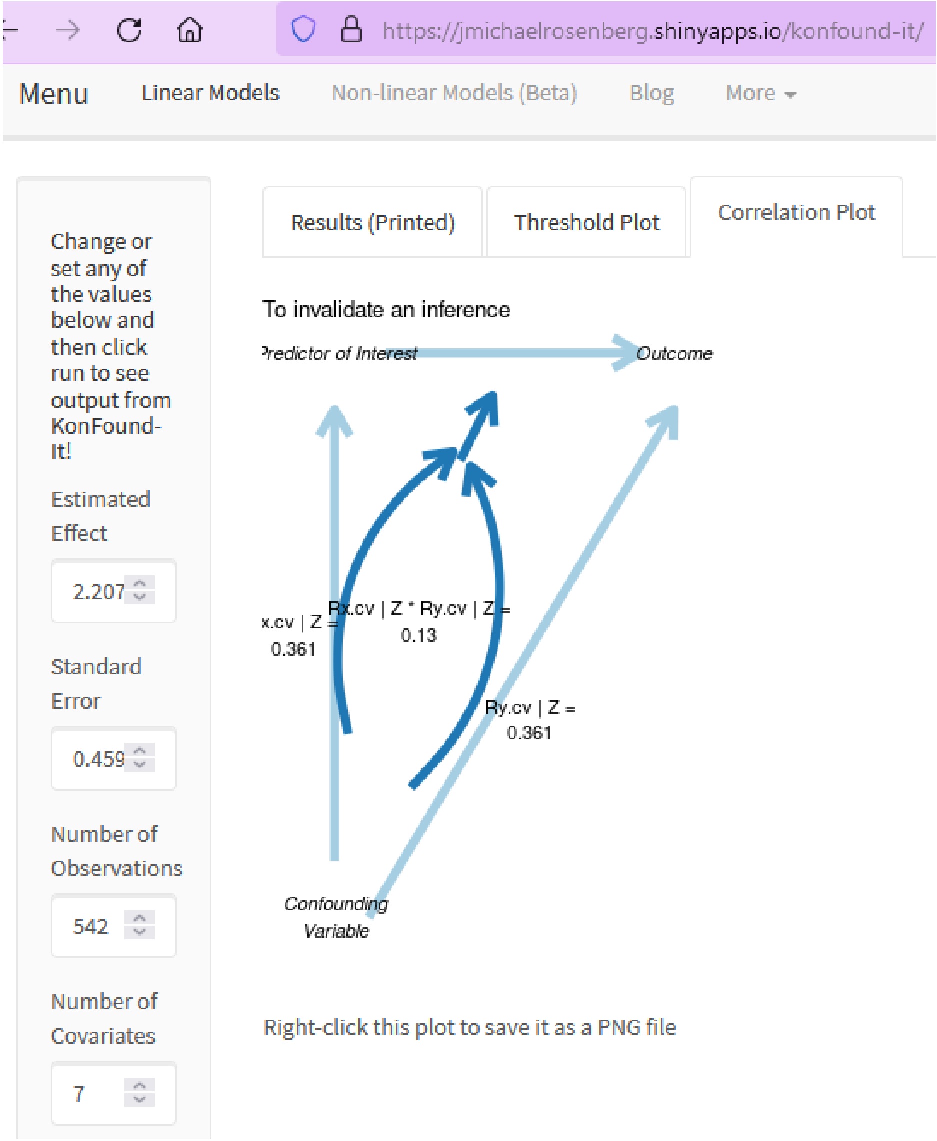

4 The National Health and Nutrition Examination Survey

We applied *treatSens, causalsens, sensemakr, EValue* and *konfound* to data from the NHANES study. As per the analysis of the BCG study, we used measured variables to represent the unmeasured confounders U. So, in effect our analyses examined the effect of not including certain confounders and we assumed that after adjustment for U and C there was no unmeasured confounding.

For *treatSens* we used Probit regression for its treatment model because X was binary, and for *causalsens* we used the one-sided confounding function because we assumed the exposure effect was the same in both exposure groups.

As this is an illustrative example of applying a QBA to unmeasured confounding, we have ignored other potential sources of bias (such as missing data) and only considered a small number of measured covariates. We restricted our analyses to participants with complete data on Y, X, C and U.

4.1 Description of the study

The NHANES study consists of a series of health and nutrition surveys conducted by the National Center for Health Statistics. Every year since 1999, approximately $5,000$ individuals of all ages are interviewed in their homes with health examinations conducted in a mobile examination centres. We analysed data from the $2015-2016$ NHANES survey [22].

Our analysis was a linear regression of systolic blood pressure (SBP) on diabetes among adults aged ≥ 18 years. Diabetes was defined as a HbA1c measurement of at least 6.5% (diabetes = 1 if HbA1C $\geq$ 6.5%, 0 otherwise) [23]. Measured covariates C were age and sex, with age as the strongest measured covariate (i.e., largest associations with diabetes and SBP). The unmeasured confounders U were BMI, ethnicity and poverty income ratio (PIR; the ratio of family income to the federal poverty line [24]). Based on the $4,576$ participants with complete data on all variables, $\hat{\beta}_{X|C}$ was 3.48 mmHg (99% CI $1.55, 5.40$ mmHg; P-value < 0.0001) and the fully adjusted estimate (i.e., adjusted for C and U) was 1.67 mmHg (99% CI $-0.27, 3.61$ mmHg; P-value 0.03). So, controlling for BMI, ethnicity and PIR explained 48% of $\hat{\beta}_{X|C}$ and resulted in a 99% CI that contained the null (i.e., P-value greater than 0.01). Statistical significance was defined at the 1% level.

Note that, we used the GitHub version of *causalsens* to enable us to set the statistical significance level to 1%. For program *EValue*, statistical

significance is fixed at 5% when using the function for a standardised mean difference. To get around this we calculated the approximate risk ratios by hand and then applied the function for a risk ratio directly to the approximate risk ratio of the CI limit of interest.

We begin with a description of the outputted results and then compare the results across the five programs.

4.2 Results

treatSens

Supplementary Figure 9(a) shows the results of the *treatSens* QBA. If the magnitudes of the diabetes$-U$ and SBP-U associations were comparable to those of the strongest measured covariate, age (diabetes$-$age $= 0.45$, and SBP$-$age $= 0.44$ on the standardised scale) then $\hat{\beta}_{X|C,U(\phi)}$ could be ≈ 0.28 standard deviations of SBP (i.e., 46% increase of $\hat{\beta}_{X|C}$ and statistically significant) or approximately 0.10 standard deviations of SBP (48% reduction of $\hat{\beta}_{X|C}$) with a P-value of 0.01. Potentially, a U comparable to age could explain away the statistical significance of $\hat{\beta}_{X|C}$. For unmeasured confounding to explain away all of $\hat{\beta}_{X|C}$ then U would need to have stronger associations with either diabetes, SBP or both (e.g., double that of diabetes$-$age ($\zeta^X \approx 1, \zeta^Y \approx 0.49$), or double that of SBP$-$age ($\zeta^X \approx 0.45, \zeta^Y \approx 1$), or in-between for both ($\zeta^X \approx 0.60, \zeta^Y \approx 0.75$)).

causalsens

Supplementary Figure 9(b) shows the results of the *causalsens* QBA, where $R^2_\alpha > 0$ corresponds to individuals in the diabetic group tending to have higher potential SBP values (to both exposure to diabetes and no exposure) than the non-diabetic group (i.e., individuals in the non-diabetic group tended to be healthier regardless of diabetic status); and the converse for $R^2_\alpha < 0$. Note that, the default scale for $R^2_\alpha > 0$ excluded the benchmark for the strongest measured covariate (age).

If the residual variance explained by U was comparable to that of the weakest measured covariate, sex, ($|R^2_\alpha| = 0.015$ or 1.5%) then $\hat{\beta}_{X|C,U(\phi)}$ could be as large as 9 mmHg or a reversed effect of about -2.5 mmHg; both with a P-value < 0.01. Additionally, if U had a partial R^2 value closer to that of age then $\hat{\beta}_{X|C,U(\phi)}$ could be ≤ -10 mmHg or ≥ 15 mmHg. A U weaker than covariate sex (with respect to proportion of the explained residual variance) could explain away the statistical significance of $\hat{\beta}_{X|C}$. For relatively weak levels of unmeasured confounding (i.e., $R^2_\alpha \approx 0.01$), then U could explain away all of $\hat{\beta}_{X|C}$ (i.e., $\hat{\beta}_{X|C,U(\phi)} = 0$).

sensemakr

The robustness values for $\hat{\beta}_{X|C}$ and 1% statistical significance were 6.65% and 3.03%, respectively. Values for $R^2_{X\sim U|C}$ and $R^2_{Y\sim U|X,C}$ exceeding 6.65% are plausible given the benchmark bounds reported in Supplementary Table 4. Therefore, we cannot exclude the possibility that unmeasured confounding could explain away all of $\hat{\beta}_{X|C}$ or all of its 1% statistical significance. This is supported by Supplementary Figures 9(c) and (d) which show that even if U was a weaker confounder than age, provided the direction of its effect was to reduce the point estimate, then accounting for U could result in a null or statistically insignificant exposure effect. Depending on the direction of the effect of U, if the magnitude of the confounding effect of U was comparable to that of age then the exposure effect could be reversed with $\hat{\beta}_{X|C,U(\phi)} = -3.41$ mmHg (Figure 9(c)) or increased to $\hat{\beta}_{X|C,U(\phi)} = 10.36$ mmHg (Supplementary Figure 10(a)).

EValue

The E-values for $\hat{\beta}_{X|C}$ and its 99% lower CI limit were 1.67 and 1.38, respectively (Supplementary Figure 11). Supplementary Table 5 reports the benchmark E-values after excluding age and sex, separately. Adjusting for age reduces the E-value for the point estimate and lower CI limit by about 1 (e.g., moved from 2.61 to 1.67). Therefore, if the confounding effect of U was comparable to age, adjusting for age, sex and U could result in E-values close to 1. Therefore, we cannot exclude the possibility that unmeasured confounding could explain away all of $\hat{\beta}_{X|C}$ or all of its 1% statistical significance.

konfound

The percent bias and impact threshold were 44.67% and 0.032, respectively. Therefore, adjusting for U could result in a statistically insignificant exposure effect if U explained away at least 44.67% of $\hat{\beta}_{X|C}$ (i.e., $\hat{\beta}_{X|C,U(\phi)} < 1.93$ mmHg; Supplementary Figure 12) or if the partial correlations of U with SBP and diabetes both exceeded 0.178 (Supplementary Figure 13). Note that, the benchmark values for $r_{X\sim U|C}$ and $r_{Y\sim U|C}$ based on age (Supplementary Table 6) were larger than 0.178 implying that a U comparable to age could explain away the statistical significance of the exposure effect.

Comparison of the results

If U was comparable to the strongest measured covariate, age, then *causalsens*, *sensemakr*, and *EValue* indicated that the exposure effect adjusted for C and U would either be null or in the reverse direction, while *treatSens* suggested

that the exposure effect would still be positive although not statistically significant at the 1% level. Program *konfound* also indicated that the statistical significance of the exposure effect was not robust to unmeasured confounding (if U was comparable to age). Given there were only two measured covariates, it seems plausible that there could be a U comparable to age (with respect to confounding of the diabetes-SBP relationship) and possibly that there were multiple unmeasured confounders. In context of multiple unmeasured confounders, then the results of *treatSens* also indicate that the exposure effect adjusted for C and U could either be null or in the reverse direction.

All programs were in line with the fully adjusted results with respect to the sensitivity of the statistical significance of the exposure effect to unmeasured confounding by BMI, ethnicity and poverty income ratio. However, the fully adjusted exposure effect remained strictly positive (i.e., unmeasured confounding by BMI, ethnicity and poverty income ratio did not explain away all of $\hat{\beta}_{X|C}$). The slight conservativeness of these programs may be because they do not account for dependencies between the multiple unmeasured confounders.

Note that, on a computer with 2.7 gigahertz the run-time of *treatSens* (with the default setting of single-threading [25]) was 10 minutes while the other programs generated their results instantaneously.

Supplementary Figure 9: Quantitative bias analysis for effect of diabetes on systolic blood pressure from the National Health and Nutrition Examination Survey. Red contour (null effect in (a) and (c), t-value at 1% significance in (d)), blue contours (bracket 1% statistically insignificant estimates), black contour or line (bias-adjusted estimates), grey shaded area (99% confidence intervals for bias-adjusted estimates), pluses, inverted triangles, crosses, and diamonds (benchmark), and black triangle (naive estimate).

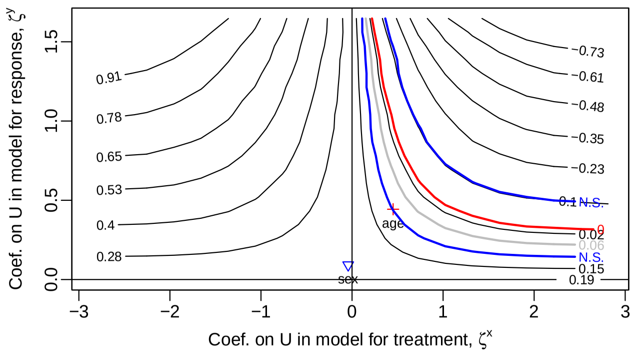

(a) *treatSens*

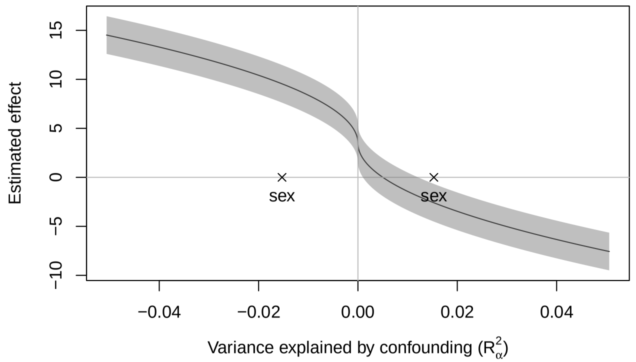

(b) *causalsens* (×: sex)

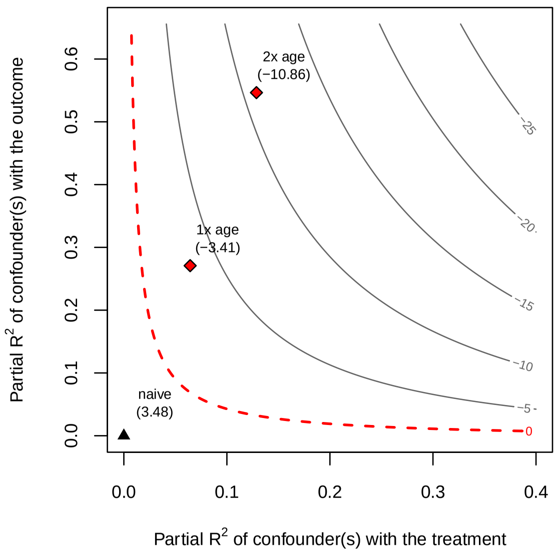

(c) *sensemakr* for point estimate

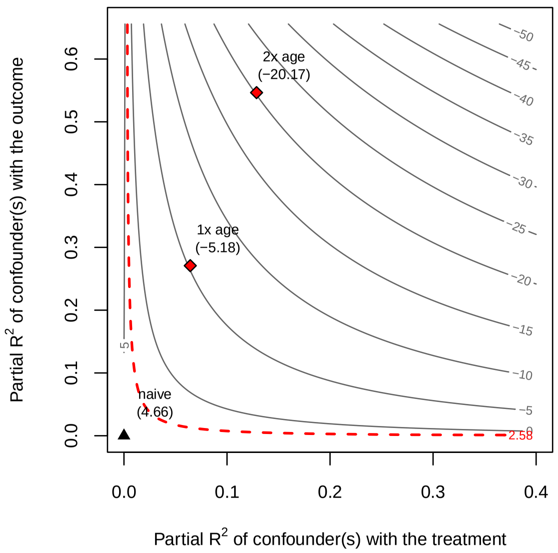

(d) *sensemakr* for t-value

Supplementary Figure 10: *sensemakr* contour plots when unmeasured confounding increases exposure effect of diabetes on systolic blood pressure: black contours (bias-adjusted estimates for point estimate in (a) and t-value at 1% significance in (b)), diamonds (benchmarks), and black triangle (naive estimate) Data from the National Health and Nutrition Examination Survey.

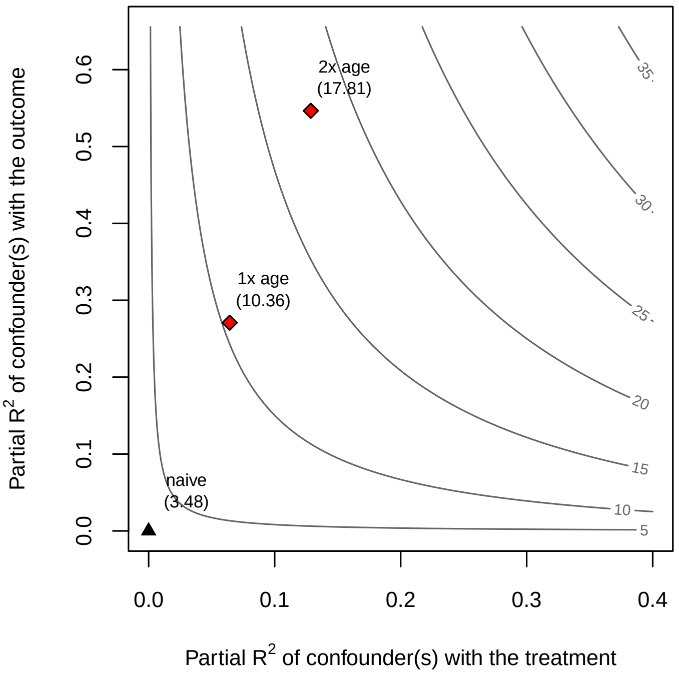

(a) *sensemakr* for point estimate

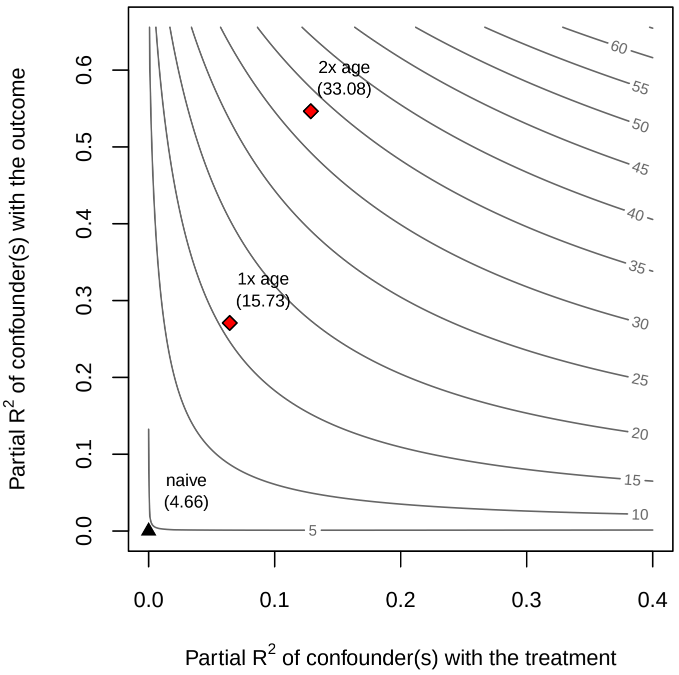

(b) *sensemakr* for t-value

Supplementary Table 4: Benchmark bounds for *sensemakr*'s bias parameters, $R^2_{X\sim U|C}$ and $R^2_{Y\sim U|X,C}$, based on partial R^2 of a measured covariate (or group of covariates) with exposure X and outcome Y. Data from the National Health and Nutrition Examination Survey.

Measured covariate	$R^2_{X\sim U\|C}(\%)$	$R^2_{Y\sim U\|X,C}(\%)$
Age	6.43	27.08
Sex	0.07	1.09
Age and sex	6.53	28.49

Supplementary Figure 11: E-value quantitative bias analysis plot for the effect of diabetes on systolic blood pressure from the National Health and Nutrition Examination Survey study. Curves denote bias parameter values that explain away 100% of the naive point estimate (red) and statistical significance at the 1% level (black); dots denote corresponding E-value.

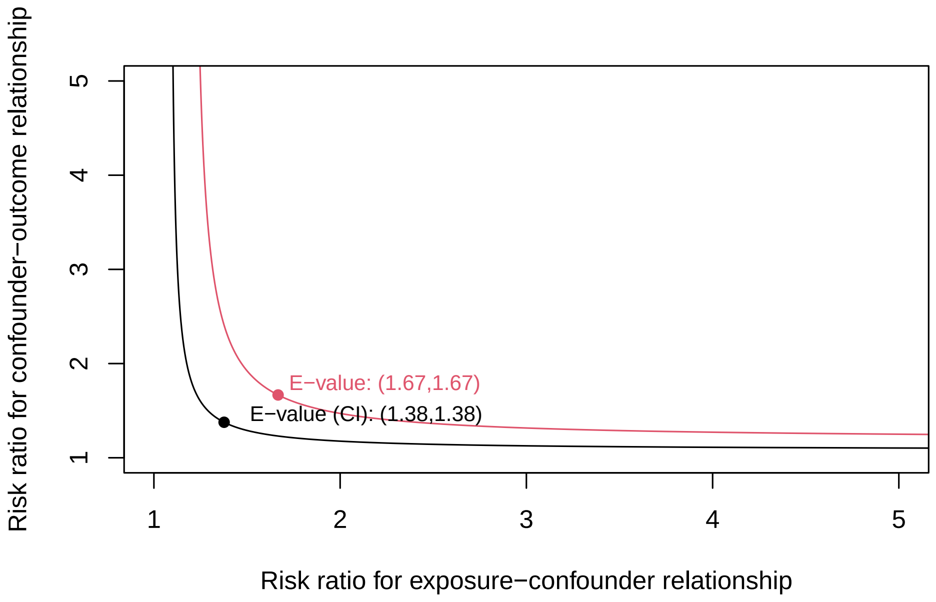

Supplementary Table 5: Benchmark E-values, for the point estimate and lower 99% confidence interval (CI) limit, based on omitting each measured covariate while controlling for the remaining measured covariates. For the effect of diabetes on systolic blood pressure from the National Health and Nutrition Examination Survey.

	E-value	
Measured covariate	Point estimate	Lower CI limit
Age	2.61	2.27
Sex	1.69	1.40

Supplementary Figure 12: *konfound*: Threshold plot representing the percent bias necessary to invalidate inference (at 1% statistical significance) for the effect of diabetes on systolic blood pressure from the National Health and Nutrition Examination Survey study.

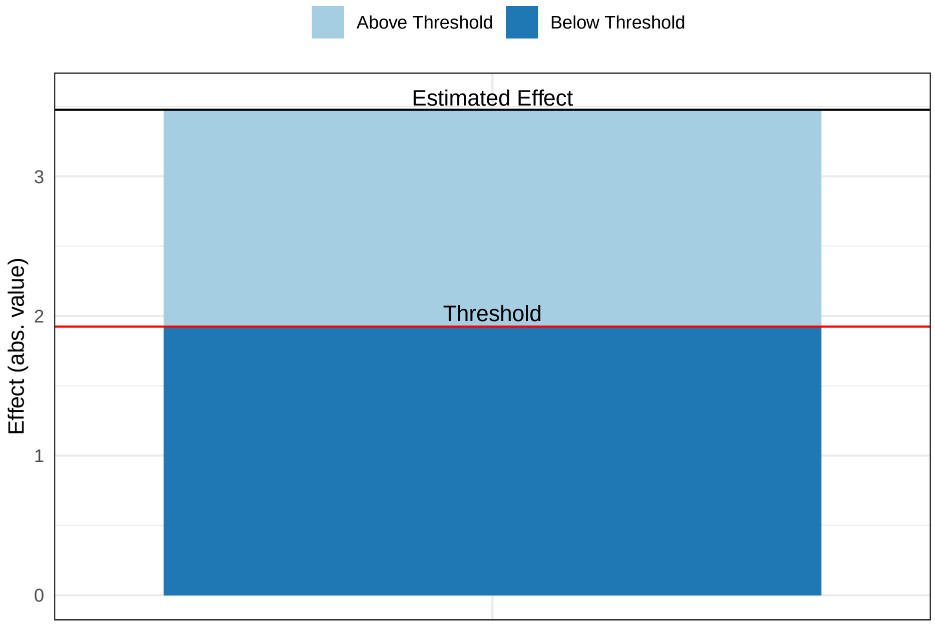

Supplementary Figure 13: *konfound*: Correlation plot of the impact of unmeasured confounding necessary to invalidate inference (at 1% statistical significance) for the effect of diabetes on systolic blood pressure from the National Health and Nutrition Examination Survey study. $r_{X \sim U|C}$ and $r_{Y \sim U|C}$ denote partial correlations between unmeasured confounder U and exposure X, and between U and outcome Y, respectively, conditional on measured covariates C.

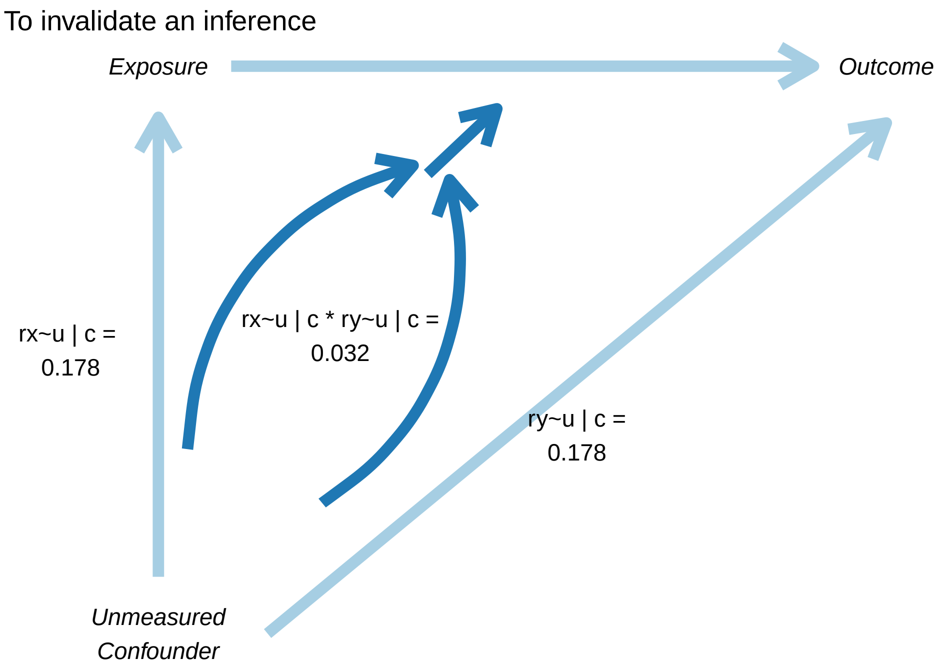

Supplementary Table 6: *konfound*: Benchmark values for $r_{X \sim U|C}$ and $r_{Y \sim U|C}$ and impact threshold $r_{X \sim U|C} \times r_{Y \sim U|C}$. Data from the National Health and Nutrition Examination Survey study.

Measured Covariate	$r_{X \sim U|C}$	$r_{Y \sim U|C}$	Impact Threshold
Age	0.25	0.46	0.12
Sex	-0.03	-0.11	-0.33

4.3 Screenshots of the web tools

This section presents screenshots of the application of the web tools of *sensemakr*, *EValue*, and *konfound* to the National Health and Nutrition Examination Survey.

Supplementary Figure 14: Screenshot of the *sensemakr* web tool applied to the National Health and Nutrition Examination Survey.

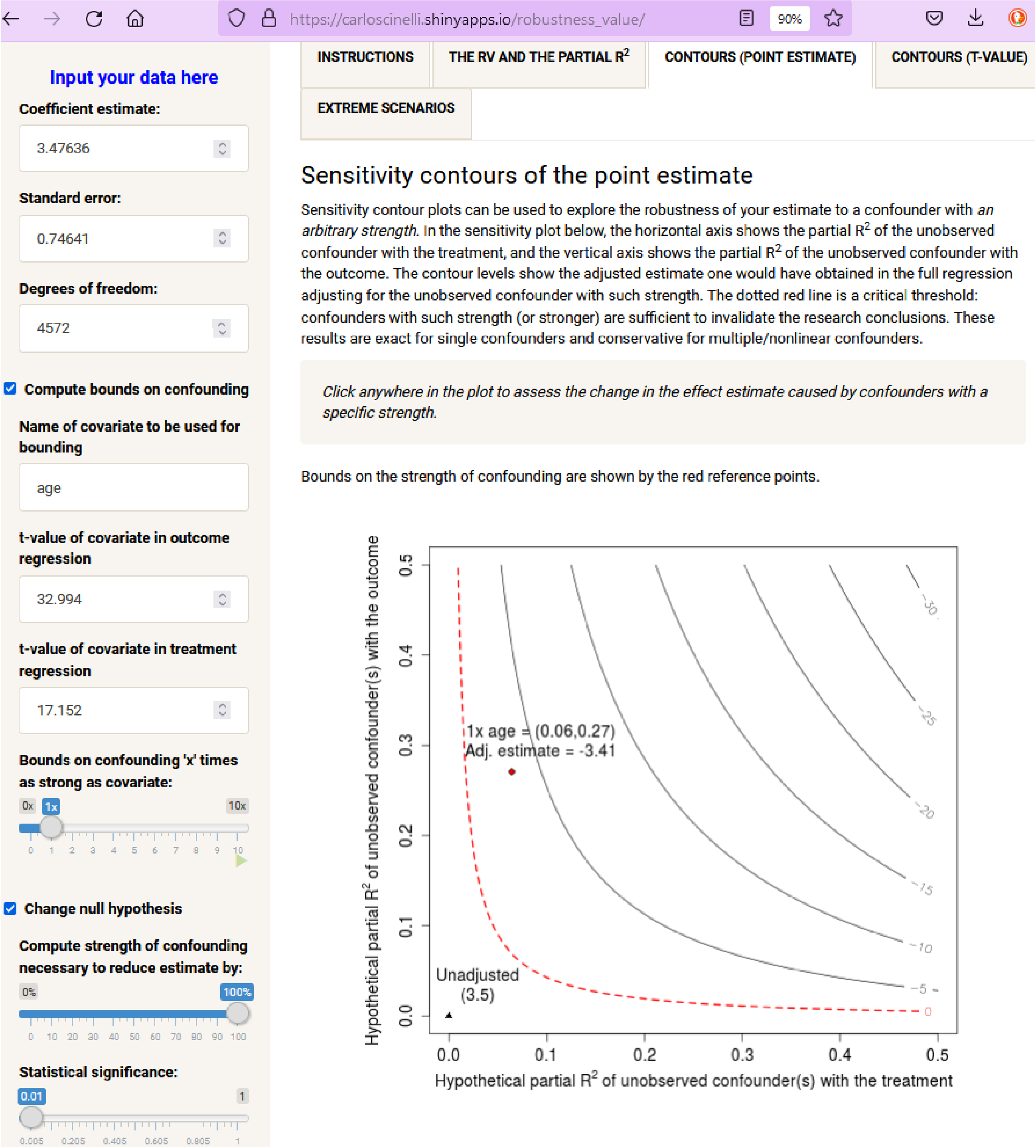

Supplementary Figure 15: Screenshot of applying the *e-value calculator* web tool to the National Health and Nutrition Examination Survey.

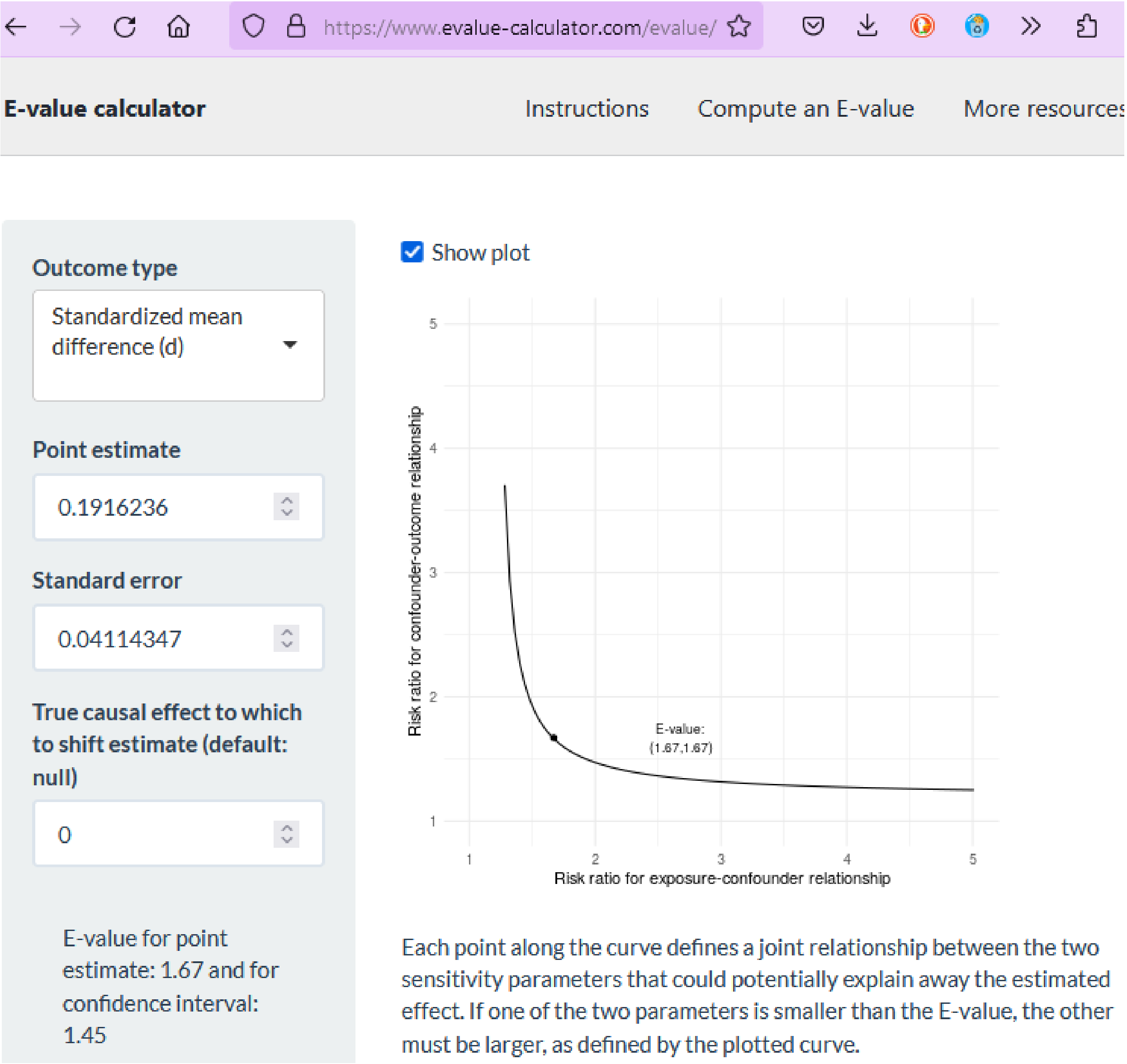

Supplementary Figure 16: Screenshot of applying the *konfound* web tool to the National Health and Nutrition Examination Survey. Note the statistical significance is fixed at the 5% level.

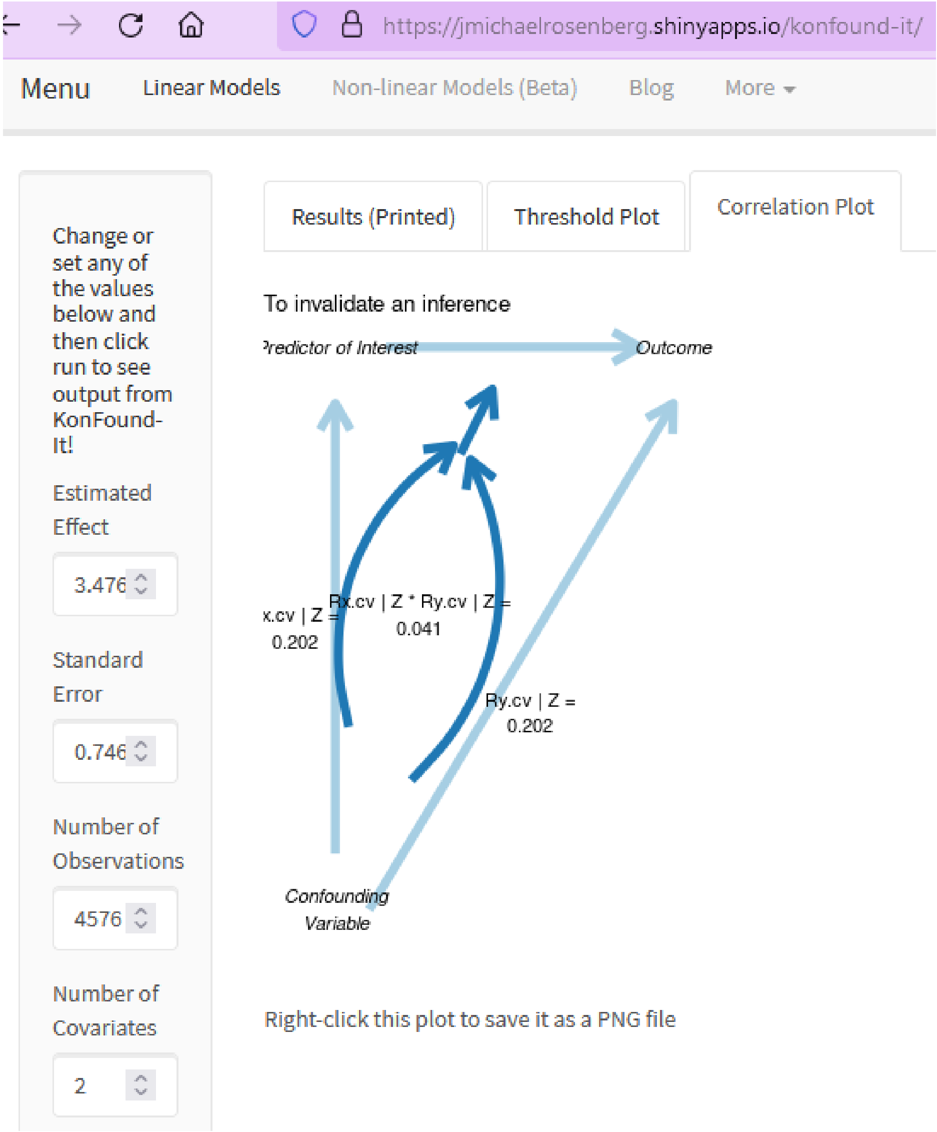

References

[1] Rubin D. Multiple imputation after 18+ years. *J Am Stat Assoc.* 1996; 91(434):473–489.

[2] Carnegie NB, Harada M, Hill JL. Assessing sensitivity to unmeasured confounding using a simulated potential confounder. *J Res Edu Eff.* 2016;9(3):395–420.

[3] Dorie V, Harada M, Carnegie NB, Hill J. A flexible, interpretable framework for assessing sensitivity to unmeasured confounding. *Stat Med.* 2016;35(20):3453–3470.

[4] Hill J, Linero A, Murray J. Bayesian Additive Regression Trees: A Review and Look Forward. *Annu Rev Stat Appl.* 2020;7:251–278.

[5] Blackwell M. A selection bias approach to sensitivity analysis for causal effects. *Polit Anal.* 2014;22(2):169–182.

[6] Rubin DB. Causal Inference Using Potential Outcomes. *J Am Stat Assoc.* 2005;100(469):322–311.

[7] Robins JM. Association, causation and marginal structural models. *Synthese.* 1999;121:151–179.

[8] Robins JM. Sensitivity analysis for selection bias and unmeasured confounding in missing data and causal inference models, Section 6–11. In: *Statistical Models in Epidemiology: The Environment and Clinical Trials*, edited by Halloran M, Berry D. Springer–Verlag: New York. 1999; .

[9] Brumback BA, Hernan MA, Haneuse SJPA, Robins JM. Sensitivity analysis for unmeasured confounding assuming a marginal structural model for repeated measures. *Stat Med.* 2004;23:749–767.

[10] Cinelli C, Hazlett C. Making sense of sensitivity: Extending omitted variable bias. *J R Stat Soc Ser B Methodol.* 2020;82(1):39–67.

[11] Zhang B, Small DS. A calibrated sensitivity analysis for matched observational studies with application to the effect of second-hand smoke exposure on blood lead levels in children. *J R Stat Soc C: Appl Stat.* 2020;69(5):1285–1305.

[12] VanderWeele TJ, Ding P. Sensitivity analysis in observational research: introducing the E-value. *Ann Intern Med.* 2017;167(4):268–274.

[13] Ding P, VanderWeele TJ. Sensitivity analysis without assumptions. *Epidemiol.* 2016;27(3):368.

[14] VanderWeele TJ. Are Greenland, Ioannidis and Poole opposed to the Cornfield conditions? A defence of the E-value. *Int J Epidemiol.* 2022; 51(2):364–371.

[15] Mathur MB, Ding P, Riddell CA, VanderWeele TJ. Website and R package for computing E-values. *Epidemiol.* 2018;29(5):e45.

[16] Mathur MB, Smith LH, Ding P, VanderWeele TJ. EValue: Sensitivity analysis for unmeasured confounding and other biases in observational studies and meta-analyses. `https://cran.r-project.org/web/packages/EValue/EValue.pdf`, (2021, accessed on 17 October 2022).

[17] Linden A, Mathur MB, VanderWeele TJ. Conducting sensitivity analysis for unmeasured confounding in observational studies using E-values: The evalue package. *SJ.* 2020;20(1):162–175.

[18] Xu R, Frank KA, Maroulis SJ, Rosenberg JM. konfound: Command to quantify robustness of causal inferences. *SJ.* 2019;19(3):523–550.

[19] Frank K, Min KS. Indices of Robustness for Sample Representation. *Sociol Methodol.* 2007;37(1):349–392.

[20] Frank KA, Maroulis SJ, Duong MQ, Kelcey BM. What would it take to change an inference? Using Rubin's causal model to interpret the robustness of causal inferences. *Educ Eval Policy Anal.* 2013;35(4):437–460.

[21] Frank KA. Impact of a confounding variable on a regression coefficient. *Sociol Methods Res.* 2000;29(2):147–194.

[22] Centers for Disease Control and Prevention/National Center for Health Statistics. National Health and Nutrition Examination Survey data. `https://wwwn.cdc.gov/nchs/nhanes/continuousnhanes/default.aspx?BeginYear=2015` (2016, accessed 17 October 2022).

[23] Geneva WHO. Use of glycated haemoglobic (HbA1c) in the diagnosis of diabetes mellitus: abbreviated report of a WHO consultation. `https://www.ncbi.nlm.nih.gov/books/NBK304267/`, (2011, accessed on 17 October 2022).

[24] Alaimo K, Briefel RR, Frongillo EA, Olson CM. Food insufficiency exists in the United States: results from the Third National Health and Nutrition Examination Survey. *Am J Public Health.* 1998;88(3):419–426.

[25] Carnegie NB, Harada M, Dorie V, Hill JL. treatSens: Sensitivity analysis for causal inference. `https://mran.microsoft.com/snapshot/2018-03-11/web/packages/treatSens/treatSens.pdf`, (2018, accessed on 17 October 2022).
