# Supplementary material for: Quantitative bias analysis in practice: review of software for regression with unmeasured confounding
Source: BMC Med Res Methodol. 2023 May 4;23:111. doi: 10.1186/s12874-023-01906-8 (PMC10158211; doi:10.1186/s12874-023-01906-8)
Supplement: Supplementary file 2 — Additional file 2. [file 12874_2023_1906_MOESM2_ESM.docx]

### R CODE USED TO APPLY PROGRAMS treatSens, causalsens, sensemakr, EValue, AND konfound TO DATA FROM THE NATIONAL HEALTH AND NUTRITION

# EXAMINATION SURVEY

# Install and load packages

install.packages(c('Hmisc', 'sensemakr', 'EValue', 'konfound', 'devtools'), dependencies=TRUE)

require(Hmisc)

require(sensemakr)

require(EValue)

require(konfound)

require(devtools)

install_github("vdorie/treatSens")

require(treatSens)

install_github("mattblackwell/causalsens", ref = "master")

require(causalsens)

#################################

########## data ##########

#################################

# Retrieve NHANES 2015-2016 data from CDC

# demographics data from https://wwwn.cdc.gov/nchs/nhanes/search/datapage.aspx?Component=Demographics&CycleBeginYear=2015

demo <- sasxport.get(file="https://wwwn.cdc.gov/Nchs/Nhanes/2015-2016/DEMO_I.XPT")

# body measures, blood pressure data from https://wwwn.cdc.gov/nchs/nhanes/search/datapage.aspx?Component=Examination&CycleBeginYear=2015

bpx <- sasxport.get(file="https://wwwn.cdc.gov/Nchs/Nhanes/2015-2016/BPX_I.XPT")

bmx <- sasxport.get(file="https://wwwn.cdc.gov/Nchs/Nhanes/2015-2016/BMX_I.XPT")

# glycohemoglobin data from https://wwwn.cdc.gov/nchs/nhanes/search/datapage.aspx?Component=Laboratory&CycleBeginYear=2015

ghb <- sasxport.get(file="https://wwwn.cdc.gov/Nchs/Nhanes/2015-2016/GHB_I.XPT")

nhanes <- merge(merge(merge(demo, bpx, by="seqn"), bmx, by="seqn"), ghb, by="seqn")

# Rename variables

colnames(nhanes)[colnames(nhanes)=='bpxsy1'] <- 'bp' #systolic blood pressure in mm Hg

colnames(nhanes)[colnames(nhanes)=='lbxgh'] <- 'HbA1c' #glycoheamoglobin or HbA1c in %

colnames(nhanes)[colnames(nhanes)=='ridageyr'] <- 'age' #age in years at screening

colnames(nhanes)[colnames(nhanes)=='riagendr'] <- 'sex' #sex: 1=male, 2=female

colnames(nhanes)[colnames(nhanes)=='indfmpir'] <- 'pir' #ratio of family income to poverty

colnames(nhanes)[colnames(nhanes)=='bmxbmi'] <- 'bmi' #body mass index to kg/m^2 to 1dp

# Create a categorical confounder "ethnicity": 1=non-Hispanic white, 2=non-Hispanic black, 3=Mexican American, 4=other

nhanes$ethnicity <- nhanes$ridreth3 #race-ethnicity: 1=Mexican American, 2=other Hispanic, 3=non-Hispanic white, 4=non-Hispanic black,

#6=non-Hispanic Asian, 7=non-Hispanic multiracial

nhanes$ethnicity[nhanes$ridreth3==1] <- 3

nhanes$ethnicity[nhanes$ridreth3==2 | nhanes$ridreth3==6 | nhanes$ridreth3==7] <- 4

nhanes$ethnicity[nhanes$ridreth3==3] <- 1

nhanes$ethnicity[nhanes$ridreth3==4] <- 2

# Create a binary exposure "diabetic": 1=diabetic, 0=not diabetic

HbA1cThreshold <- 6.5

nhanes$diabetic <- vector(length=nrow(nhanes)) #diabetic: 0=not diabetic, 1=diabetic

nhanes$diabetic[nhanes$HbA1c>=HbA1cThreshold] <- 1

# Delete rows whose age<18 years and NA in one of the variables required for the analysis

nhanes <- nhanes[nhanes$age>=18 & rowSums(is.na(nhanes[,c('bp', 'HbA1c', 'diabetic', 'age', 'sex', 'bmi', 'ethnicity', 'pir')]))==0, c('bp', 'HbA1c', 'diabetic',

'age', 'sex', 'bmi', 'ethnicity', 'pir')]

nhanes <- data.frame(lapply(nhanes, function(x) as.numeric((x))))

# Write to csv

write.csv(nhanes[,c('bp', 'diabetic', 'age', 'sex', 'bmi', 'ethnicity', 'pir')], 'NhanesData.csv', row.names=F)

######################################

########## naive analysis ##########

######################################

# Fit linear regression assuming no unmeasured confounding

NhanesNaiveModel <- lm(bp~diabetic+sex+age, data=nhanes)

summary(NhanesNaiveModel)

confint(NhanesNaiveModel, level=0.99)

#####################################################

########## analysis adjusted for C and U ##########

####################################################

# Fit linear regression adjusting for C and U

NhanesAdjustedeModel <- lm(bp~diabetic+sex+age+bmi+pir+factor(ethnicity), data=nhanes)

summary(NhanesAdjustedeModel)

confint(NhanesAdjustedeModel, level=0.99)

######################################

########## treatSens ##########

######################################

# Perform treatSens

NhanesTreatSens <- treatSens(formula=bp~diabetic+sex+age, resp.family=gaussian, trt.family=binomial(link='probit'), data=nhanes)

# Produce a contour plot

sensPlot(NhanesTreatSens, signif.level=0.01, txtlab=TRUE)

######################################

########## causalsens ##########

######################################

# Perform causalsens

NhanesCausalsens <- causalsens(model.y=NhanesNaiveModel, model.t=glm(diabetic~sex+age, family=binomial('logit'), data=nhanes), cov.form=~sex+age,

data=nhanes, level=0.99)

# Produce a line plot

plot(NhanesCausalsens, type='r.squared')

######################################

########## sensemakr ##########

######################################

# Perform sensemakr using individual-level data

summary(sensemakr(formula=bp~diabetic+sex+age, data=nhanes, treatment='diabetic', benchmark_covariates=list(sex='sex', age='age', "(age +

sex)"=c('sex', 'age')), alpha=0.01))

# Perform sensemakr using summary statistics

summary(sensemakr(estimate=summary(NhanesNaiveModel)$coef['diabetic','Estimate'], se=summary(NhanesNaiveModel)$coef['diabetic','Std. Error'],

dof=NhanesNaiveModel$df, alpha=0.01))

# Produce a contour plot for the point estimate, bias towards the null

ovb_contour_plot(model=NhanesNaiveModel, treatment='diabetic', benchmark_covariates=list(age='age'), kd=1:2, sensitivity.of='estimate')

# Produce a contour plot for the t-value, bias towards the null

ovb_contour_plot(model=NhanesNaiveModel, treatment='diabetic', benchmark_covariates=list(age='age'), kd=1:2, sensitivity.of='t-value',

t.threshold=qt(0.995, NhanesNaiveModel$df))

# Produce a contour plot for the point estimate, bias away from the null

ovb_contour_plot(model=NhanesNaiveModel, treatment='diabetic', benchmark_covariates=list(age='age'), kd=1:2, sensitivity.of='estimate', reduce=F)

# Produce a contour plot for the t-value, bias away from the null

ovb_contour_plot(model=NhanesNaiveModel, treatment='diabetic', benchmark_covariates=list(age='age'), kd=1:2, sensitivity.of='t-value',

t.threshold=qt(0.995, NhanesNaiveModel$df), reduce=F)

######################################

########## EValue ##########

######################################

# Perform E-value using linear regression results at the 5% significance level

NhanesEvalues <- evalues.OLS(est=summary(NhanesNaiveModel)$coef['diabetic', 'Estimate'], se=summary(NhanesNaiveModel)$coef['diabetic',

'Std. Error'], sd=sd(nhanes$bp))

# Perform E-value using standardised linear regression results at the 5% significance level

evalues.MD(est=summary(NhanesNaiveModel)$coef['diabetic', 'Estimate']/sd(nhanes$bp), se=summary(NhanesNaiveModel)$coef['diabetic',

'Std. Error']/sd(nhanes$bp))

# Produce a line plot for E-value

bias_plot(NhanesEvalues['RR','point'], xmax=5)

# Manually calculate the 99% CI

# calculate the standardised mean difference

MD <- summary(NhanesNaiveModel)$coef['diabetic', 'Estimate']/sd(nhanes$bp)

# transform the mean difference to risk ratio using the approximation given in Table 2 of VanderWeele and Ding 2017

RR <- exp(0.91*MD)

# similarly, calculate the lower and upper CI limits of the mean difference and transform them to risk ratios

MD_LL <- confint(NhanesNaiveModel, level=0.99)['diabetic','0.5 %']/sd(nhanes$bp)

RR_LL <- exp(0.91*MD_LL)

MD_UL <- confint(NhanesNaiveModel, level=0.99)['diabetic','99.5 %']/sd(nhanes$bp)

RR_UL <- exp(0.91*MD_UL)

# calculate the E-value and 99% CI

Evalues <- evalues.RR(est=RR, lo=RR_LL, hi=RR_UL)

# Manually calculate benchmark E-values

# calculate the E-value based on omitting sex while adjusting for age

evalues.OLS(est=summary(lm(bp~diabetic+age, data=nhanes))$coef['diabetic', 'Estimate'], se=summary(lm(bp~diabetic+age, data=nhanes))$coef['diabetic',

'Std. Error'], sd=sd(nhanes$bp))

# calculate the E-value based on omitting age while adjusting for sex

evalues.OLS(est=summary(lm(bp~diabetic+sex, data=nhanes))$coef['diabetic', 'Estimate'], se=summary(lm(bp~diabetic+sex, data=nhanes))$coef['diabetic',

'Std. Error'], sd=sd(nhanes$bp))

######################################

########## konfound ##########

######################################

# Perform konfound using individual-level data

konfound(model_object=NhanesNaiveModel, tested_variable=diabetic, alpha=0.01, to_return=c('print', 'raw_output'))

# Perform konfound using summary statistics

pkonfound(est_eff=summary(NhanesNaiveModel)$coef['diabetic','Estimate'], std_err=summary(NhanesNaiveModel)$coef['diabetic','Std. Error'],

n_obs=nrow(nhanes), n_covariates=summary(NhanesNaiveModel)$df[1], alpha=0.01, to_return=c('print', 'raw_output'))

# Produce a threshold plot and a correlation plot using individual-level data

konfound(model_object=NhanesNaiveModel, tested_variable=diabetic, alpha=0.01, to_return=c('thresh_plot', 'corr_plot'))

# Manually calculate benchmark partial correlations

cor(nhanes$diabetic, nhanes$age)

cor(nhanes$bp, nhanes$age)

cor(nhanes$diabetic, nhanes$sex)

cor(nhanes$bp, nhanes$sex)
