# Supplementary material for: Quantitative bias analysis in practice: review of software for regression with unmeasured confounding
Source: BMC Med Res Methodol. 2023 May 4;23:111. doi: 10.1186/s12874-023-01906-8 (PMC10158211; doi:10.1186/s12874-023-01906-8)
Supplement: Supplementary file 3 — Additional file 3. [file 12874_2023_1906_MOESM3_ESM.docx]

/***********************************************************************************************

STATA CODE USED TO APPLY PROGRAMS sensemakr, evalue, AND konfound TO

DATA FROM THE NATIONAL HEALTH AND NUTRITION EXAMINATION SURVEY

***********************************************************************************************/

/********************************

INSTALL USER-WRITTEN COMMANDS

********************************/

* INSTALL sensemakr

ssc install sensemakr

* INSTALL evalue

findit evalue // CLICK ON BLUE HYPERLINKS TO INSTALL

* INSTALL konfound

ssc install moss

ssc install indeplist

ssc install matsort

findit konfound // CLICK ON BLUE HYPERLINKS TO INSTALL

/*************************************************************************

IMPORT DATASET - ALREADY PROCESSED AND EXPORTED BY OUR SUPPLIED R-CODE

*************************************************************************/

import delimited NhanesData.csv, clear

/****************

NAIVE ANALYSIS

*****************/

regress bp diabetic sex age

/*******************************

ANALYSIS ADJUSTED FOR C AND U

********************************/

regress bp diabetic sex age bmi pir i.ethnicity

/***************

SENSEMAKR

****************/

* ROBUSTNESS VALUES AND BENCHMARK BOUNDS

sensemakr bp diabetic sex age, treat(diabetic) alpha(0.01) benchmark(sex age) ///

gbenchmark(sex age) kd(1)

* CONTOUR PLOT FOR THE POINT ESTIMATE; BIAS TOWARDS THE NULL

sensemakr bp diabetic sex age, treat(diabetic) alpha(0.01) benchmark(age) kd(1 2) contourplot

* CONTOUR PLOT FOR THE T-VALUE; BIAS TOWARDS THE NULL

sensemakr bp diabetic sex age, treat(diabetic) alpha(0.01) benchmark(age) kd(1 2) tcontourplot

* CONTOUR PLOT FOR THE POINT ESTIMATE; BIAS AWAY FROM THE NULL

sensemakr bp diabetic sex age, treat(diabetic) alpha(0.01) benchmark(age) kd(1 2) ///

contourplot noreduce

* CONTOUR PLOT FOR THE T-VALUE; BIAS AWAY FROM THE NULL

sensemakr bp diabetic sex age, treat(diabetic) alpha(0.01) benchmark(age) kd(1 2) ///

tcontourplot noreduce

/***************

EVALUE

****************/

* CALCULATE STANDARDISED MEAN DIFFERENCE (SMD)

summarize bp

gen std_bp = bp/r(sd)

regress std_bp diabetic sex age, level(99)

local smd_estimate = _b[diabetic]

local smd_lci = _b[diabetic] - invttail(e(df_r),0.005)*_se[diabetic]

* CALCULATE APPROXIMATE RISK RATIO FOR POINT ESTIMATE AND LOWER CONFIDENCE INTERVAL

local RR_estimate = exp(0.91*`smd_estimate')

local RR_lci = exp(0.91*`smd_lci')

* E-VALUE FOR THE POINT ESTIMATE AND LOWER CONFIDENCE INTERVAL

evalue rr `RR_estimate', lcl(`RR_lci') figure

* BENCHMARK E-VALUES FOR THE POINT ESTIMATE AND LOWER CONFIDENCE INTERVAL

* OMITTING COVARIATE AGE

regress std_bp diabetic sex, level(99)

local smd_estimate = _b[diabetic]

local smd_lci = _b[diabetic] - invttail(e(df_r),0.005)*_se[diabetic]

local RR_estimate = exp(0.91*`smd_estimate')

local RR_lci = exp(0.91*`smd_lci')

evalue rr `RR_estimate', lcl(`RR_lci')

* OMITTING COVARIATE SEX

regress std_bp diabetic age, level(99)

local smd_estimate = _b[diabetic]

local smd_lci = _b[diabetic] - invttail(e(df_r),0.005)*_se[diabetic]

local RR_estimate = exp(0.91*`smd_estimate')

local RR_lci = exp(0.91*`smd_lci')

evalue rr `RR_estimate', lcl(`RR_lci')

/***************

KONFOUND

****************/

* PERCENT BIAS, IMPACT THRESHOLD, BENCHMARK VALUES AND GRAPHICAL PLOT FOR PERCENT BIAS

regress bp diabetic sex age, level(99)

konfound diabetic, sig(0.01)
